# Local brain connectome parameters across the spectrum of clinical cognitive decline

**DOI:** 10.3389/fnins.2026.1840382

**Published:** 2026-06-19

**Authors:** Demet Yüksel Dal, Boran A. Kilic, Gurur Gamgam

**Affiliations:** 1 Electrical and Electronics Engineering Department, Fatih Sultan Mehmet Vakif University, İstanbul, Türkiye; 2 Electrical and Electronics Engineering Department, Boğaziçi University, İstanbul, Türkiye

**Keywords:** Alzheimer's disease, brain connectome, dMRI, fMRI, structural and functional network

## Abstract

Neurological disorders such as Alzheimer's disease, Parkinson's disease, and autism disrupt the brain's structural and functional organization, particularly in specific regions, and ultimately lead to cognitive impairments. In Alzheimer's disease-related dementia, neuronal degeneration impairs structural connectivity between brain regions, which in turn leads to functional breakdowns. This phenomenon, referred to as disconnection syndrome, manifests as connectivity breakdowns in affected regions, with these localized changes indirectly influencing the entire brain network. As the disease progresses, patterns consistent with compensatory-type reorganization have been described in the literature, accompanied by structural and functional changes that have been hypothesized to transiently mitigate cognitive decline during early stages. This study examines the structural and functional reorganization of the brain across the clinical spectrum of Alzheimer's disease by analyzing local nodal changes using measures such as degree, strength, clustering coefficient, and betweenness centrality. Our findings show that early-stage nodal patterns are consistent with this hypothesized reorganization, whereas later-stage changes are dominated by progressive structural decline alongside persistent functional reorganization. Because the present study is cross-sectional and group-level, the compensatory interpretation should be regarded as a working hypothesis rather than a confirmed mechanism, and these exploratory patterns require validation in independent and longitudinal cohorts before clinical translation.

## 1 Introduction

Neurodegenerative diseases are characterized by the gradual degradation of brain cells and loss of neurons (Yu et al., 2021). These pathological changes have adverse effects on structural connections between brain regions, potentially leading to observed degradation in functional connections. Such changes have been observed in Alzheimer's Disease Dementia (ADD) cases, together with the accumulation of two abnormal proteins, beta-amyloid and tau (Hanseeuw et al., 2019). They lead to mild to severe cognitive impairment across the spectrum of dementia. ADD cases account for 60 − 80% of all cases of dementia, causing immense economic and social burden (Gaugler et al., 2022). Although no cure has been found to reverse this degeneration, it is widely accepted that lifestyle has a significant impact on the pace of progress in dementia, if not the prevention (Chowdhary et al., 2022). Hence, prognosis and early diagnosis of ADD can facilitate timely intervention to lifestyle choices easing the aforementioned burden on society and individuals. Furthermore, it would enable assessing the outcome of clinical trials of treatments by monitoring the progress of dementia.

Brain connectomes have emerged as an effective and systematic approach to studying the aforementioned structural and functional connections (*network edges*) between brain regions (*network nodes*), most notably in the cortical regions (Sporns, 2013). The associated field of connectomics is built upon the graph theory and exploits the graph characterization metrics to discover and understand the changes in connectomes in neurodegenerative diseases, including ADD.

A large part of the current literature on brain connectomes is based on studies of global changes in the brain connectomes measured by global network measures. Despite the high sensitivity reported in neurodegenerative diseases, global measures cannot capture local changes that may manifest ADD at different stages through local neural degeneration and regeneration. Such changes are likely to be captured at different topological scales. For instance, there are cases where normal cognitive function is reported despite an amyloid plaque deposition, the foremost physiological biomarker of ADD, suggesting that the brain may be locally resistant to the disease (Yıldrım et al., 2023).

Although connectome datasets provide a unique view point to study the brain as a structure of interconnected and communicating components, these studies have a number of pitfalls and challenges. Graph theoretical analyses of both nodal and global measures in Alzheimer's disease have produced inconsistent findings, largely due to methodological variations in connectome construction. Tijms et al. and Dal et al. have reported that these inconsistent findings in the literature can be attributed to differences in imaging modalities such as functional MRI (fMRI), diffusion MRI (dMRI) and electroencephalogram (EEG), atlas templates, methods for defining connectivity edges (e.g., correlation vs. tractography), or connectivity types (binary vs. weighted) (Tijms et al., 2013; Yuksel Dal et al., 2024). While the use of EEG recordings provide a temporal dimension at the cost of spatial resolution, the most common two modalities are dMRI for structural networks (sNET) construction and fMRI for functional networks (fNET) construction. The spatial, topological and temporal scale variations add a further complexity to connectome models (Betzel and Bassett, 2017). Topological scale choice determines whether the whole brain and subnetworks are considered. Spatial scale determines the scale of the smallest units (*nodes*) used, ranging from individual voxels to cortical parcellations (Kaiser, 2011).

Neurodegenerative diseases do not represent discrete or binary states; instead, they unfold gradually, with both symptoms and neural degeneration progressing over time. Correspondingly, changes in the brain connectome are likely to be dynamic rather than stationary, given that the brain is an exceptionally adaptive organ. Therefore, connectome-based studies must account for this temporal dimension to avoid seemingly inconsistent findings, which can otherwise hinder the development of a holistic understanding of how these diseases affect brain organization. For example, in fMRI studies comparing ADD patients with Healthy Controls (HCs), (Xiang et al. 2013) reported a decrease in the global clustering coefficient and an increase in path length when using partial correlation, suggesting reduced network interconnectedness. In contrast, (Sanz-Arigita et al. 2010) observed no significant change in the global clustering coefficient and a decrease in average path length when applying synchronization likelihood, suggesting that the brain may adapt by shortening pathways between regions to preserve efficient communication.

In dMRI studies, (Fischer et al. 2015) and (Lo et al. 2010) reported that structural networks in ADD cases exhibit a higher average path length but an unchanged clustering coefficient using the Harvard-Oxford and Automated Anatomical Labelling (AAL) atlases, respectively. This suggests that while long-range connectivity is compromised, local connectivity remains relatively preserved. In contrast, (Pereira et al. 2018) and (Wang et al. 2016) found both an increased average path length and a higher clustering coefficient in ADD cases relative to healthy controls, employing the Desikan-Killiany and AAL atlases. The elevated clustering coefficient in these studies likely indicates altered local connectivity, potentially reflecting compensatory reorganization or disrupted network efficiency in response to ADD.

These findings collectively suggest that there are alterations in structural and functional connectivity patterns in individuals with ADD compared to HCs. The inconsistencies in findings highlight the complexity of understanding structural and functional connectivity changes in neurodegenerative disorders such as ADD and highlight the importance of considering various factors and examining local connections when interpreting connectivity data.

Despite growing evidence supporting network measures as promising candidate biomarkers of cognitive decline, multimodal nodal alterations across the full clinical spectrum remain insufficiently characterized. The novelty of the present study lies in the systematic nodal comparison of multiple graph metrics—degree, strength, clustering coefficient, and betweenness centrality—across both structural and functional connectomes spanning the SCIMCIADD spectrum, together with their evaluation in a low-dimensional classification framework. Neurodegenerative processes evolve gradually rather than as discrete states, and recent connectome studies have suggested that low-dimensional representations may better capture this progressive reorganization (Bayr et al., 2025). To address this gap, we investigate nodal changes in both structural and functional networks using Schaefer's 400-node cortical atlas (Schaefer et al., 2017). We also examine the potential of these nodal metrics as candidate biomarkers across successive stages of cognitive decline, namely from subjective cognitive impairment (SCI) to mild cognitive impairment (MCI), and from MCI to Alzheimer's disease dementia (ADD).

The remainder of this paper is organized as follows. Section 2 describes the data acquisition, connectome construction, nodal measurements, and the statistical and classification methods used. Section 3 details the experiments, including a multi-scale statistical analysis and the final classification results. Finally, Section 4 discusses the impact of different scales on the discriminative power of network measures, examines localized changes at various disease stages, and provides concluding remarks and recommendations for using nodal measures as diagnostic biomarkers in brain connectome research.

## 2 Methodology

**Figure 1:**
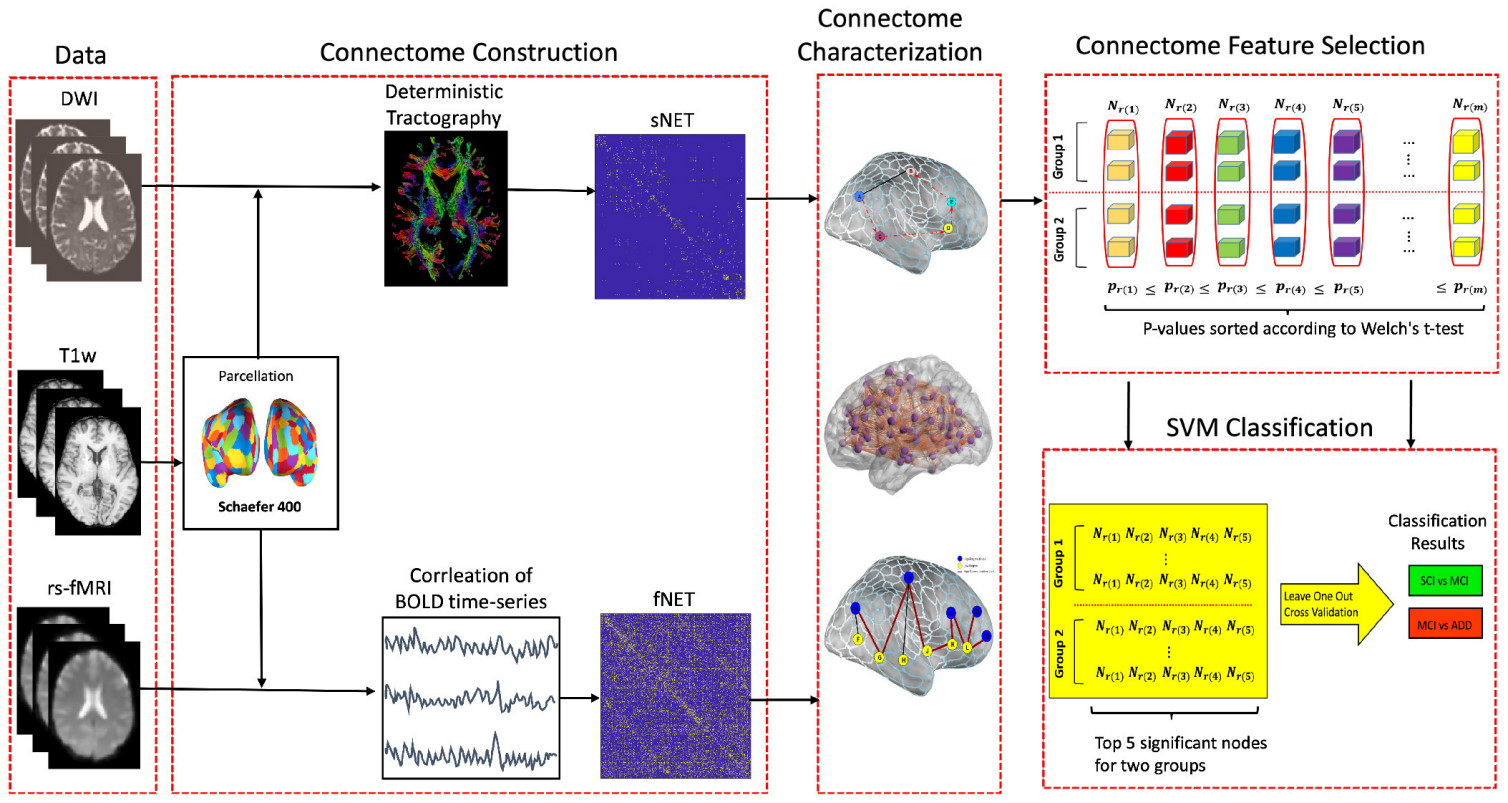
Block diagram of the methodological framework for local brain connectome analysis across cognitive decline. The pipeline summarizes multimodal image acquisition, connectome construction, nodal graph analysis, feature selection, and classification across the SCI-MCI and MCI-ADD stages.

This study outlines a methodological framework to investigate local brain connectome alterations across the clinical spectrum of cognitive decline. A block diagram illustrating the complete analysis pipeline is shown in Figure 1. Neuroimaging data acquisition includes DWI, T1-weighted (T1w), T2-weighted (T2w), and resting-state functional MRI (rs-fMRI). Structural parcellation of the T1w images is performed using the Schaefer-400 cortical atlas, which segments the cortex into 400 regions of interest (ROIs) (Schaefer et al., 2017). sNETs are generated via deterministic tractography based on DWI data, while fNETs are constructed using parcel-wise summary BOLD signals derived from rs-fMRI. Functional connectivity is estimated using regularized partial correlation coefficients obtained through elastic-net regression. Detailed procedures for the construction of sNETs and fNETs are presented in subsequent sections.

To characterize local connectivity patterns, both structural and functional networks are analyzed using graph-theoretical nodal metrics, including degree, strength, clustering coefficient, and betweenness centrality. These nodal measures serve as region-specific features representing local connectivity profiles. For group differentiation—specifically, SCI vs. MCI, and MCI vs. ADD—a feature selection strategy is employed to identify the most discriminative ROIs based on their nodal measurement values. By conducting this analysis at the 400-parcel resolution of the Schaefer atlas, the study aims to identify spatially localized connectivity alterations associated with progressive stages of cognitive decline.

### 2.1 Participants and data acquisition

A total of 88 participants were enrolled in the study following the provision of written informed consent, in accordance with ethical approvals obtained from the Istanbul Faculty of Medicine Ethics Committee^1^ and the Boğaziçi University Ethics Committee.^2^ Table 1 summarizes the demographic and clinical characteristics of the study cohort. All MRI scans were performed using a Philips Achieva 3T MRI scanner (Philips Medical Systems, The Netherlands) equipped with a 32-channel head coil, located at the Neuroimaging Unit of the Hulusi Behet Life Sciences Research Laboratory, Istanbul Faculty of Medicine.

**Table 1:** Summary of the cohort of participants.

Characteristic	SCI (*n* = 24)	MCI (*n* = 46)	ADD (*n* = 18)
Age	56.95 ± 7.54	62.04 ± 9.78	69.83 ± 8.52
Gender (F/M)	12/12	23/23	8/10
CI-FCRST	0.88 ± 0.10	0.68 ± 0.15	0.36 ± 0.21
TFR-FCRST	33.45 ± 3.85	20.63 ± 5.46	6.33 ± 7.23

CI-FCRST: Cued Index score of the Free and Cued Selective Reminding Test, reflecting memory performance under semantic cueing conditions.

TFR-FCRST: Total Free Recall score of the Free and Cued Selective Reminding Test, reflecting unaided episodic retrieval.

Clinical assessments were conducted in accordance with the National Institute on Aging and Alzheimer's Association (NIA-AA) research framework (McKhann et al., 2011; Albert et al., 2011; Jessen et al., 2014; Jack et al., 2018). We note that classification in the present cohort was based on clinical and neuropsychological criteria rather than amyloid or tau biomarker confirmation; cerebrospinal fluid (CSF) and positron emission tomography (PET) Amyloid, Tau, and Neurodegeneration (ATN) biomarkers were not available for these participants. The cohort therefore reflects clinically defined probable AD-spectrum impairment across all three groups, and not biomarker-confirmed Alzheimer's disease. Individuals with a clinical cognitive impairment diagnosis at any stage based on NIA-AA criteria or clinical cognitive tests and their relatives without any cognitive impairment (CI) symptoms, individuals with no CI symptoms and no relatives with any CI diagnosis, provided that they had not started (or changed the dosage of) neurological medication within the last 3 months are included in the study. The individuals with a history of current or past neurological or psychiatric disorders adversely affecting cognition, alcohol or substance abuse, major head trauma with loss of consciousness, and those who had white matter hyperintensities on MRI with a Fazekas score of 2 and 3, were excluded from the study. Contraindications for scanning at MRI were another exclusion criterion from our study. The clinical diagnosis of amnestic type MCI is based on identifying an objective deficit in episodic memory with a total free recall score (TFR) of 27 or lower and a Cue Index (CI) of 0.67 or lower on the Free and Cued Selective Reminding Test (FCSRT) (Buschke, 1973). Participants scoring above 27 in TFR-FCSRT or above 0.67 in CI-FCSRT were classified as SCI. The chosen TFR cutoff of 27 was more liberal than 24, a highly sensitive predictor of future dementia in dementia-free individuals (Grober et al., 2010). ADD participants met NIA-AA criteria for probable AD with an amnestic presentation (McKhann et al., 2011). SCI subjects were project volunteers responding to advertisements, scoring at least 1 on either the Cognitive Functions Instrument – Subject form (CFI-S) or CFI – Study Partner form (CFI-SP) (Li et al., 2017). Comprehensive neurological and neuropsychological examinations, along with cranial MRI, confirmed SCI, MCI, and ADD diagnoses by a panel of behavioral neurologists. The panel ensured that ADD participants had a Clinical Dementia Rating (CDR) score of 0.5 or 1, indicating very mild or mild dementia; MCI patients had a CDR score of 0.5 and CDR-Sum of Boxes (CDR-SOB) score of 0.5 or 1; and SCI subjects had a CDR score of 0. SCI therefore serves as the earliest clinical reference group within the scope of this study, rather than as a cognitively normal control group. During clinical recruitment, special care was taken to differentiate AD-spectrum decline from other dementia syndromes, including Lewy body dementia, vascular cognitive impairment, and frontotemporal degeneration; participants meeting criteria for any non-AD etiology were excluded prior to enrolment.

Based on these assessments, participants were classified into three diagnostic groups: Alzheimer's disease dementia (ADD, *n* = 18), amnestic-type MCI (*n* = 46), and subjective cognitive impairment (SCI, *n* = 24). Importantly, no cognitively normal control group was included in this study.

### 2.2 Brain network construction

All imaging modalities were co-registered to an isotropic resolution of 1.5mm using a custom multimodal preprocessing pipeline that integrates open-source toolboxes, including FreeSurfer, FSL, and TORTOISE. This pipeline was applied to co-register T1-weighted MRI, T2-weighted MRI, fMRI, and DWI volumes for each participant, with alignment quality subsequently verified by experienced neurologists. Cortical parcellation was performed on the T1-weighted volumes using the Schaefer atlas at 400-parcel resolution (Schaefer et al., 2017). Each parcel was further assigned to one of seven canonical Yeo functional networks: visual, somatomotor, dorsal attention, salience/ventral attention, limbic, frontoparietal, and default (Yeo et al., 2011). No global signal regression (GSR) or WM/CSF nuisance regression was applied prior to functional connectivity estimation. The effect of GSR on resting-state connectivity remains debated: while it can suppress physiological artifacts, it also introduces artificial negative correlations and can impose group-differential confounds when physiological noise profiles differ systematically between diagnostic groups, a realistic concern in aging and dementia cohorts (Fox et al., 2005; Liu et al., 2017; Power et al., 2014; Ciric et al., 2017). Retaining the global signal is therefore a defensible choice in this setting (Liu et al., 2017), and the use of elastic-net regularized partial correlation (Li et al., 2019) provides an alternative means of controlling for shared parcel-wise variance without explicit mean-signal subtraction. We nevertheless acknowledge that the absence of WM/CSF nuisance regression, motion scrubbing, and archived framewise displacement or DVARS summaries represents a preprocessing limitation. In the absence of these confound controls, functional connectivity estimates may retain residual motion and physiological noise, which could differentially affect diagnostic groups. The fNET findings should therefore be interpreted as exploratory, and preprocessing sensitivity is discussed further in Section 4 and assessed in Supplementary Section 2.3.

DTI was reconstructed^3^ from multi-shell (b-values: 0 − 3000s/mm^2^) DWI data using the dual tensor basis solution to the Stejskal-Tanner equations (Westin et al., 2002). DWI was reconstructed using fourth order Runge-Kutta method with 0.15 minimum fractional anisotropy (FA), 0.7mm stepsize, 20mm minimum fiber length and 35° maximum angle as a curvature threshold (Tench et al., 2002). sNET edge weights between nodes *i* and *j* (cortical parcels) are determined by


$$\begin{array}{l} e_{ij}=\frac{2}{V_{i}+V_{j}}\underset{k}{\sum }W_{ik}W_{jk}\;, \end{array}$$ (1)

where, *e*_*ij*_ represents the normalized weighted edge, *V*_*i*_ and *V*_*j*_ represent volumes in *mm*^3^ and *W*_*ik*_ quantifying the association between the *i*-th parcel and the *k*-th fiber as described in (Durusoy et al. 2021). sNETs were thresholded by removing all edges with a normalized weight (Equation 1) below 0.01, a value consistent with established practice for excluding spurious low-reproducibility connections in volume-normalized structural connectomes (Tsai, 2018) (see Supplementary Section 2.1 for details). Functional networks were constructed from resting-state fMRI data using regularized partial correlation coefficients estimated via the elastic net, in order to address the limitations of standard partial correlation approaches (Lim et al., 2019).

fNETs were constructed from rs-fMRI data by estimating partial correlation coefficients using an elastic net regularization (Lim et al., 2019). Specifically, Singular Value Decomposition (SVD) was applied to the BOLD signals extracted from each region, yielding a summary signal that encapsulates both the temporal dynamics and spatial distribution of neural activity (Bowman et al., 2012). Subsequently, elastic net regularization, which combines both ℓ_1_ (LASSO) and ℓ_2_ (Ridge) penalties, was applied to control for overfitting and enhance model generalizability. The regularization parameters were optimized using a grid search strategy to achieve an optimal trade-off between sparsity and coefficient stability. Following the estimation of the regularized coefficients, partial correlation values were derived using a symmetric criterion that incorporates both the magnitude and sign of the regression coefficients. This approach mitigates the limitations associated with conventional partial correlation analysis and provides robust estimates of functional connectivity. Full elastic-net parameter settings, the lambda and alpha grids, the standardization procedure, and the handling of negative edges are reported in Supplementary Section 2.2.

### 2.3 Graph measures

Following the construction of multi-modal sNETs and fNETs, network measures were computed to extract insights for both scientific and clinical applications. The adoption of “complex network theory,” which originated from statistical physics research and has seen widespread applications across various fields over the past decade is particularly prevalent in brain research (Barabási and Albert, 1999; Watts and Strogatz, 1998; Bullmore and Sporns, 2009). This theory enables researchers to quantitatively compare brain networks across different individuals and even within the same individual over time, offering a valuable tool for understanding the dynamic nature of brain connectivity. In this study, we utilized the Brain Connectivity Toolbox (BCT) (Rubinov and Sporns, 2010) to compute key graph-theoretical metrics, including degree, strength, clustering coefficient, and betweenness centrality. Among the many available measures, a specific subset was selected for this study based on their relevance and frequent use in brain connectivity studies, especially in studies related to ADD.

The degree is the number of connections a node has with other nodes in the network. In the context of brain networks, a node's degree reflects how interconnected a particular brain region is with other regions. This metric is particularly useful for understanding the role and influence of individual brain regions in the overall network structure. A high degree suggests a brain region plays a significant role in communication and information flow, while a lower degree might indicate a more specialized or isolated function. Node degree is formulated as


$$\begin{array}{l} d_{i}=\overset{N}{\underset{j=1}{\sum }}a_{ij}\;, \end{array}$$ (2)

where *d*_*i*_ denotes the degree of node *i*, *N* is the total number of nodes in the network, and *a*_*ij*_ is the binarized form of *w*_*ij*_, indicating the presence or absence of a connection between nodes *i* and *j*. Specifically, *a*_*ij*_ = 1 if a link between nodes *i* and *j* exists, and *a*_*ij*_ = 0 otherwise. Additionally, self-connections are excluded, i.e., *a*_*ii*_ = 0 for all *i*.

Strength is the total weight of connections linked to a specific node. It is a fundamental measure in network analysis, providing insights into the connectivity and centrality of individual nodes within the network. Measuring connection strength between brain regions helps understand information flow pathways and their efficiency in transmitting information. Additionally, altered connection strengths might be linked to the development or progression of neurological disorders. Node strength is calculated as


$$\begin{array}{l} s_{i}=\overset{N}{\underset{j=1}{\sum }}w_{ij}\;, \end{array}$$ (3)

where *w*_*ij*_ denotes the weight of the connection between nodes *i* and *j* and *N* is the total number of nodes in the network.

The clustering coefficient quantifies the degree of interconnectedness or clustering among the immediate neighbors of a node within a network. It measures the likelihood that the neighbors of a particular node are also connected. A higher clustering coefficient indicates a greater amount of localized communication and information processing within specific brain regions, while a lower clustering coefficient suggests a more distributed or less specialized organization of brain regions in their interactions and communication. The clustering coefficient of node *i*, *C*_*i*_, is expressed by (Watts and Strogatz 1998) through the following formula, as


$$\begin{array}{l} C_{i}=\frac{2T_{i}}{d_{i}\left(d_{i}-1\right)}, \end{array}$$ (4)

where *T*_*i*_ is the number of triangles associated with node *i*. *T*_*i*_ is calculated as


$$\begin{array}{l} T_{i}=\underset{j,k}{\sum }a_{ij}\cdot a_{jk}\cdot a_{ki}, \end{array}$$ (5)

where *a*_*ij*_ indicates whether there is a connection (1) or no connection (0) between node *i* and node *j*. *a*_*jk*_ indicates whether there is a connection (1) or no connection (0) between node *j* and node *k*. *a*_*ki*_ indicates whether there is a connection (1) or no connection (0) between node *k* and node *i*.

Betweenness centrality quantifies the extent to which a node serves as a bridge along the shortest paths between other nodes in the network. This metric is crucial for identifying key nodes that connect or mediate within the network. An increase in betweenness centrality indicates that a node is becoming a critical connection point in the brain network, facilitating communication between various brain regions as information flows through it more frequently. Conversely, a decrease in betweenness centrality suggests a decline in the node's role as a communication hub, potentially leading to a diminishing significance as a bridge for information exchange within the brain graph. Betweenness centrality of node *i*, *B*_*i*_, is defined by the following formula (Freeman, 1978):


$$\begin{array}{l} B_{i}=\underset{s\neq i\neq t}{\sum }\frac{\sigma_{st}\left(i\right)}{\sigma_{st}} \end{array}$$ (6)

where σ_*st*_ represents the total number of shortest paths from node *s* to node *t*, and σ_*st*_(*i*) denotes the number of shortest paths from node *s* to node *t* that pass through node *i*.

### 2.4 Mass univariate statistical analysis and classification framework

To disentangle the local topological drivers of cognitive decline, we adopted an exploratory, hypothesis-generating statistical framework. Our objective was to identify candidate local biomarkers with high discriminative potential rather than to confirm a global network hypothesis. This analysis proceeded in two stages: a mass univariate statistical characterization of individual nodes, followed by a multivariate classification using Support Vector Machines (SVM).

#### 2.4.1 Feature definition and statistical characterization

Let = {1, …, *N*} denote the set of all participants, divided into diagnostic subgroups _1_ and _2_ (e.g., SCI vs. MCI). For each participant *j*∈ and each node *i*∈{1, …, *M*} (where *M* = 400 corresponds to the Schaefer atlas parcels), we extracted a topological feature vector. Let $x_{i}^{\left(j\right)}$ represent a specific nodal metric (e.g., degree, strength) for the *i*-th node of the *j*-th participant.

We employed a Mass Univariate Analysis approach to evaluate the discriminative power of each node individually. For a given node *i*, we tested the null hypothesis *H*_0, *i*_:μ_*i*, 1_ = μ_*i*, 2_ against the alternative that the group means are unequal. The test statistic *t*_*i*_ was computed using Welch's *t*-test to account for unequal variances and sample sizes between the clinical cohorts:


$$\begin{array}{l} t_{i}=\frac{{\bar{x}}_{i,1}-{\bar{x}}_{i,2}}{\sqrt{\frac{s_{i,1}^{2}}{n_{1}}+\frac{s_{i,2}^{2}}{n_{2}}}} \end{array}$$ (7)

where ${\bar{x}}_{i,g}$ and $s_{i,g}^{2}$ denote the sample mean and unbiased variance of the metric for node *i* in group *g*, and *n*_*g*_ is the group sample size.

#### 2.4.2 Statistical thresholding and correction strategy

In this study, we assessed statistical significance based on a Per-Comparison Error Rate (PCER) threshold of α = 0.05. We deliberately opted against standard Family-Wise Error Rate (FWER) corrections (Bonferroni Correction) for two critical methodological reasons:

1. Non-Independence of Tests: Standard correction methods assume that the *M* simultaneous tests are statistically independent. However, brain network nodes exhibit intrinsic topological modularity and high spatial autocorrelation. Consequently, the feature set **X** = {*x*_1_, …, *x*_*M*_} contains significant covariance, meaning the Effective Number of Independent Tests (*M*_*eff*_) is strictly less than the total number of nodes (*M*_*eff*_≪*M*). Treating these partially correlated biological units as orthogonal experiments would result in an overly conservative penalty.
2. Exploratory Sensitivity: Given the subtle nature of network reorganization in early-stage cognitive decline, prioritizing Specificity (controlling false positives) via strict FWER correction excessively inflates the Type II error rate (False Negatives). To avoid discarding potentially biologically relevant candidate biomarkers in this hypothesis-generating phase, we prioritized Sensitivity, reporting nodes that satisfy the individual condition *P*(|*T*|>|*t*_*i*_|) < 0.05.

Our analytical framework follows a two-stage biomarker discovery paradigm (Dosenbach et al., 2010; Woo et al., 2017): (1) exploratory univariate screening to identify candidate features, followed by (2) confirmatory multivariate validation via cross-validated prediction. In this design, the initial screening phase prioritizes sensitivity to avoid discarding potentially meaningful signals, while the classification stage provides rigorous statistical control. Specifically, our 1,000-iteration permutation test evaluates whether the top-5 node set— selected via the univariate ranking—supports above-chance classification. This approach implicitly corrects for multiple comparisons at the model level: even if individual node *p*-values include false positives, nodes lacking true discriminative signal will not improve prediction accuracy beyond the permutation-based null distribution. Therefore, the permutation test, not the univariate *p*-values, constitutes the primary inferential gate in our framework.

#### 2.4.3 Feature selection and SVM classification

Following the univariate characterization, we ranked all *M* nodes according to their absolute t-statistic |*t*_*i*_|, which serves as a proxy for discriminative power. To reduce dimensionality and isolate the most salient network drivers, we constructed low-dimensional feature vectors using the top *K* = 5 most significant nodes.

These selected features served as inputs to a linear Support Vector Machine (SVM) classifier. The generalization performance of the classifier was evaluated using Leave-One-Out Cross-Validation (LOOCV). At each iteration, the model was trained on *N*−1 subjects and evaluated on the held-out subject. To ensure a leakage-free evaluation, feature ranking and top-*K* node selection were repeated independently within each training fold. This low-dimensional, node-centered classification framework is consistent with recent local connectome-based machine learning studies reporting that compact sets of discriminative regional features can support robust classification across neurological disorders (Chelef et al., 2026; Yuksel Dal et al., 2024).

To assess whether classification performance depends on classifier choice or primarily on the selected features, we additionally evaluated an L2-regularized logistic regression model under identical LOOCV and within-fold feature selection procedures; results are reported in Supplementary Table S17.

#### 2.4.4 Null model validation

To verify that the reported classification accuracies reflect genuine neuropathological signals rather than spurious correlations arising from high-dimensional feature selection, we conducted a non-parametric permutation test with 1,000 iterations. Critically, in each iteration the diagnostic labels *Y* = {*y*^(1)^, …, *y*^(*N*)^} were randomly shuffled prior to any analysis, and the complete pipeline was re-executed from scratch: this included Welch's *t*-test-based node ranking, top-K feature selection, and LOOCV-based SVM classification. This design ensures that the node selection step itself—the stage most susceptible to data leakage in high-dimensional settings—is fully embedded within the permutation loop. Consequently, any node that appears discriminative solely due to chance will also appear discriminative under shuffled labels, and will not yield above-chance classification. The empirical null distribution thus obtained provides a stringent, leakage-free baseline against which the true classification accuracy is compared. The statistical significance of the observed accuracy was determined by its position relative to this null distribution (*p* < 0.05, two-tailed).

## 3 Results

The exploration of key nodal network measures—nodal degree, strength, clustering coefficient, and betweenness centrality,—aims to capture distinct aspects of connectivity changes during clinical cognitive decline. Examining these measures independently across both sNET and fNET provides deeper insights into how specific network components respond as the disease advances from SCI to MCI and ultimately to ADD.

**Figure 2:**
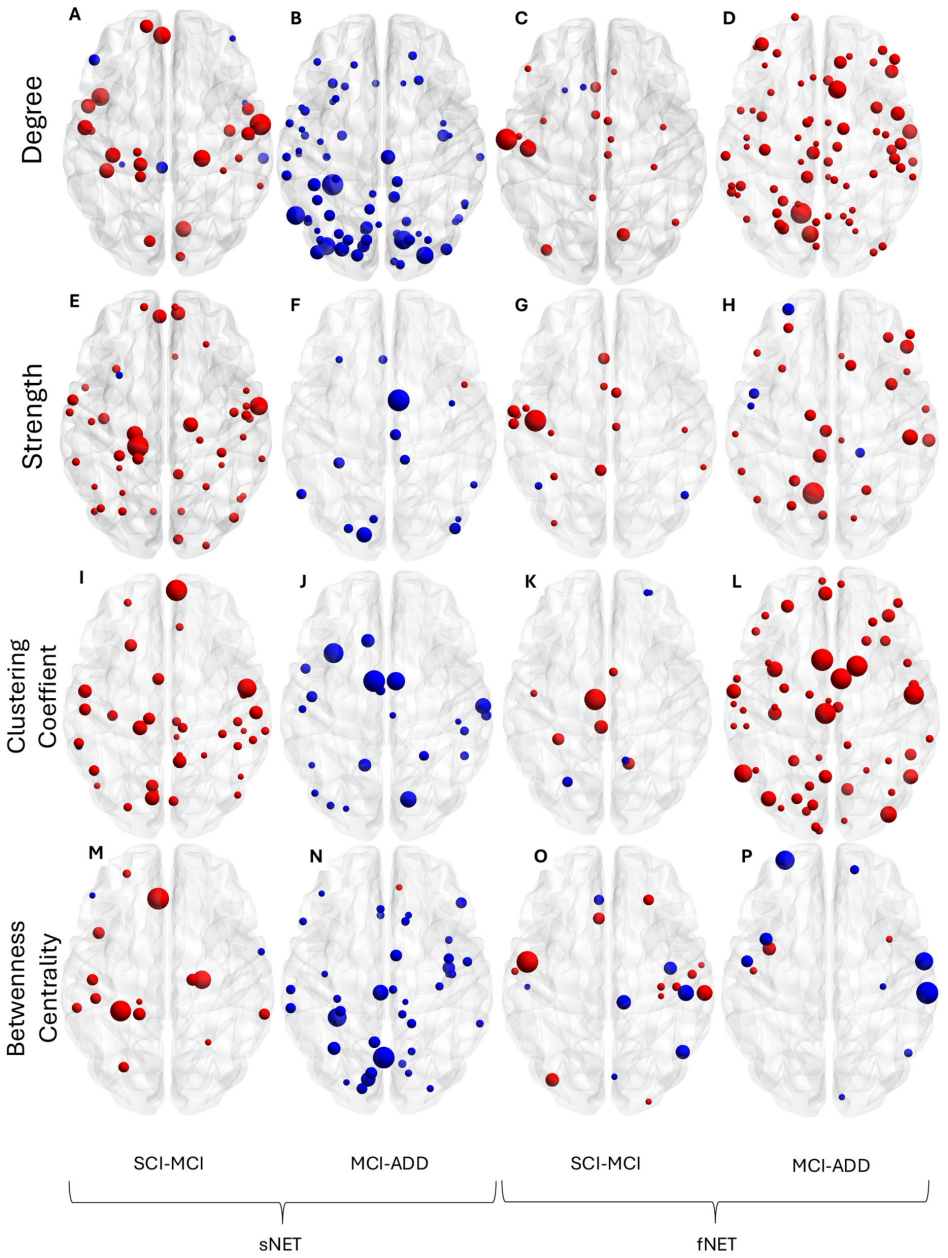
Cortical maps of statistically significant nodal alterations across stages of cognitive decline. Only parcels with significant between-group differences (Welch's *t*-test, *p* < 0.05, uncorrected) are shown. Marker size is inversely proportional to the *p*-value, such that larger markers indicate stronger statistical significance. Marker color encodes the direction of the group difference: red denotes a higher value in the later-stage group (i.e., MCI > SCI, or ADD > MCI), while blue denotes a lower value in the later-stage group (i.e., MCI < SCI, or ADD < MCI). Rows correspond to the four nodal graph metrics (**A–D** degree, **E–H** strength, **I–L** clustering coefficient, **M–P** betweenness centrality); columns correspond to the two disease transitions (SCI-MCI and MCI-ADD) and two network modalities (sNET and fNET). The maps highlight regionally specific alterations and reveal a stage-dependent reversal pattern that is particularly pronounced in structural networks, where early-stage increases give way to widespread decreases in the MCI-to-ADD transition.

Each measure provides distinct theoretical insights into both local and global connectivity patterns, contributing uniquely to the comprehensive analysis of network structure. By systematically evaluating these measures, the analysis identifies those measures most capable of elucidating network resilience, vulnerability, and adaptability, providing a nuanced understanding of how various brain network components respond to the progression of neurodegeneration. An overview of the spatial distribution of statistically significant nodal alterations across all evaluated graph measures is presented in Figure 2.

### 3.1 Nodal degree

Nodal degree (Equation 2), representing the number of connections associated with each node, was analyzed to identify alterations in network connectivity across stages of cognitive decline. Figures 2A–D presents the differential nodal degree maps derived from Welch's *t*-test (Equation 7), highlighting cortical regions with statistically significant changes. In the SCI-vs.-MCI comparison, 29 significant nodes (*p* < 0.05) were identified among the 400 nodes in the sNETs (Figure 2A), whereas 21 significant nodes were detected in the fNETs (Figure 2C). In the MCI-vs.-ADD comparison, the number of significant nodes increased to 66 in the sNETs (Figure 2B) and 71 in the fNETs (Figure 2D) (*p* < 0.05).

The maps reveal a stage-dependent and modality-specific pattern. In the sNET, the SCI-vs.-MCI comparison was characterized predominantly by increased nodal degree, whereas the MCI-vs.-ADD comparison was dominated by decreases, suggesting a transition from early structural compensation to later disconnection. In contrast, the fNET showed predominantly increased nodal degree in both comparisons, with this pattern becoming more widespread in the MCI-vs.-ADD stage.

**Figure 3:**
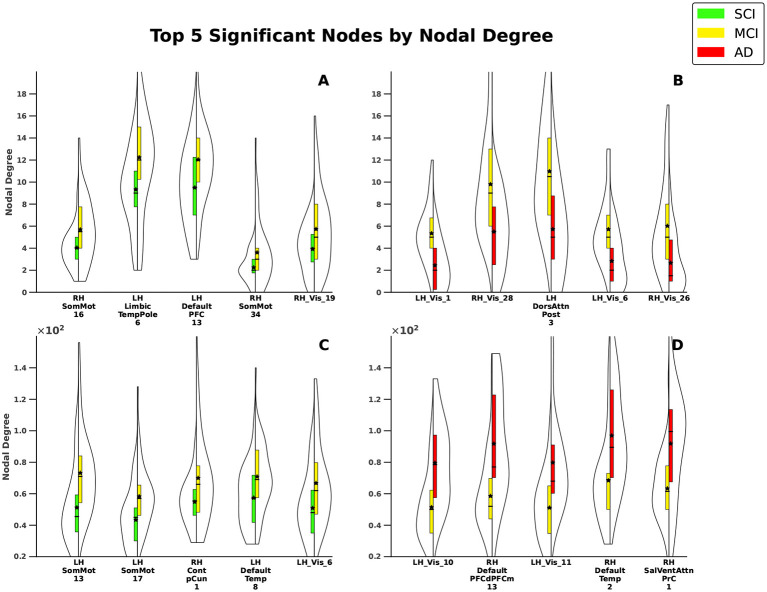
Distributions of nodal degree for the top five most discriminative nodes in structural (sNET, top row) and functional (fNET, bottom row) networks across the cognitive decline spectrum. **(A)** Top five most discriminative sNET nodes for the SCI vs. MCI comparison (*p* < 0.011). A statistically significant increase in nodal degree is observed in the transition from SCI (green) to MCI (yellow). **(B)** Top five most discriminative sNET nodes for the MCI vs. ADD comparison (*p* < 0.001). A statistically significant decrease is observed as the disease progresses from MCI (yellow) to ADD (red). **(C)** Top five most discriminative fNET nodes for the SCI vs. MCI comparison (*p* < 0.011). Similar to the sNET, a statistically significant increase in nodal degree is observed in the transition from SCI (green) to MCI (yellow). **(D)** Top five most discriminative fNET nodes for the MCI vs. ADD comparison (*p* < 0.003). Unlike the sNET, a continued statistically significant increase is observed from MCI (yellow) to ADD (red). (*) and (-) inside colored bars represent the mean and median of the distributions, respectively.

Figure 3 shows the distribution of the nodal degree for the top five most discriminative sNET nodes. A significant increase in nodal degree is observed in the transition from SCI to MCI (*p* < 0.011), as shown in Figure 3A. In contrast, a statistically significant decrease is observed in the transition from MCI to ADD for the most discriminative nodes in that comparison (*p* < 0.001), as illustrated in Figure 3B. Figures 3C, D report the identical statistical analysis for fNETs. Similar to sNETs, Figure 3C reveals a statistically significant increase in the nodal degree of selected fNET nodes as cognitive decline increases from SCI to MCI (*p* < 0.011). Unlike the sNET, the top five fNET nodes with the highest discriminative power for the MCI-vs.-ADD classification show a continued increase in nodal degree from MCI to ADD (*p* < 0.001), as shown in Figure 3D.

### 3.2 Nodal strength

Nodal strength (Equation 3), which measures the cumulative weight of connections associated with each node, was analyzed to identify alterations in weighted network connectivity across stages of cognitive decline. Figures 2E–H presents the differential nodal strength maps derived from Welch's *t*-test, highlighting cortical regions with statistically significant changes. In the SCI-vs.-MCI comparison, 45 significant nodes (*p* < 0.05) were identified among the 400 nodes in the sNETs (Figure 2E), whereas 17 significant nodes were detected in the fNETs (Figure 2G). In the MCI-vs.-ADD comparison, the number of significant nodes decreased to 15 in the sNETs (Figure 2F) but increased to 30 in the fNETs (Figure 2H) (*p* < 0.05).

The maps reveal a stage-dependent and modality-specific pattern. In the sNET, the SCI-vs.-MCI comparison was characterized predominantly by increased nodal strength, whereas the MCI-vs.-ADD comparison was dominated by decreases, suggesting a transition from early structural compensation to later weakening of weighted connectivity. In contrast, the fNET showed predominantly increased nodal strength in both comparisons, although a small number of decreases were also observed, particularly in the later stage.

**Figure 4:**
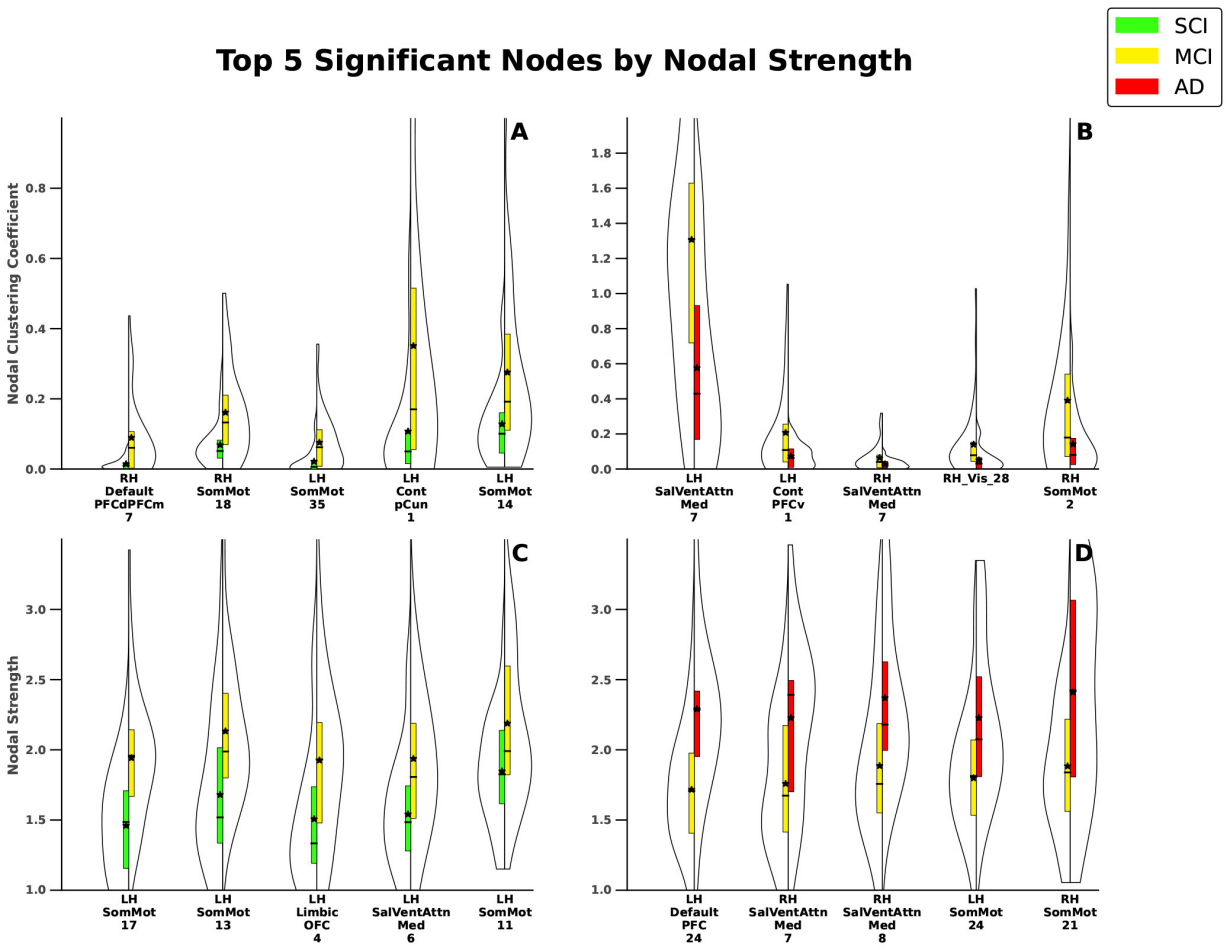
Distributions of nodal strength for the top five most discriminative nodes in structural (sNET, top row) and functional (fNET, bottom row) networks across the cognitive decline spectrum. **(A)** Top five most discriminative sNET nodes for the SCI vs. MCI comparison (*p* < 0.001). A statistically significant increase in nodal strength is observed in the transition from SCI (green) to MCI (yellow). **(B)** Top five most discriminative sNET nodes for the MCI vs. ADD comparison (*p* < 0.008). In contrast, a statistically significant decrease is observed as the disease progresses from MCI (yellow) to ADD (red). **(C)** Top five most discriminative fNET nodes for the SCI vs. MCI comparison (*p* < 0.006). A statistically significant increase in nodal strength is observed in the transition from SCI (green) to MCI (yellow). **(D)** Top five most discriminative fNET nodes for the MCI vs. ADD comparison (*p* < 0.001). Unlike the sNET, a continued statistically significant increase in nodal strength is observed throughout the disease progression from MCI (yellow) to ADD (red). Notably, the sets of top discriminating nodes for the SCI vs. MCI and MCI vs. ADD classifications are mutually exclusive across both network types. (*) and (-) inside colored bars represent the mean and median of the distributions, respectively.

Examining the top five most discriminative nodes for each comparison provided further detail. For sNETs, a significant increase in nodal strength was observed as cognitive decline progressed from SCI to MCI (*p* < 0.001), as depicted in Figure 4A. This trend reversed during the transition to the second stage of the disease, with a significant decrease in nodal strength from MCI to ADD (*p* < 0.008), as shown in Figure 4B. For fNETs, a statistically significant increase in the nodal strength of selected nodes was also observed from SCI to MCI (*p* < 0.006), as illustrated in Figure 4C. However, unlike in the structural network, the top five fNET nodes with the highest discriminative power for the MCI-vs.-ADD classification showed a continued, significant increase in strength from MCI to ADD (*p* < 0.001), which is shown in Figure 4D. Notably, for both sNET and fNET, the sets of top-discriminating nodes for the SCI-vs.-MCI and MCI-vs.-ADD classifications were mutually exclusive.

### 3.3 Nodal clustering coefficient

The nodal clustering coefficient (Equations 4 and 5), reflecting the degree of local interconnectivity among neighboring nodes, was analyzed to identify alterations in local network organization across stages of cognitive decline. Figures 2I–L presents the differential nodal clustering coefficient maps derived from Welch's *t*-test, highlighting cortical regions with statistically significant changes. In the SCI-vs.-MCI comparison, 36 significant nodes (*p* < 0.05) were identified among the 400 nodes in the sNETs (Figure 2I), whereas only 10 significant nodes were detected in the fNETs (Figure 2K). In the MCI-vs.-ADD comparison, 21 significant nodes were identified in the sNETs (Figure 2J) and 56 in the fNETs (Figure 2L) (*p* < 0.05).

The maps reveal a stage-dependent and modality-specific pattern. In the sNET, the SCI-vs.-MCI comparison was characterized predominantly by increased clustering coefficient, whereas the MCI-vs.-ADD comparison was dominated by decreases, suggesting a shift from early local reorganization to later structural disintegration. In contrast, the fNET showed a mildly increased pattern in the SCI-vs.-MCI comparison and a more widespread increase in the MCI-vs.-ADD comparison, indicating persistent local functional segregation as the disease advances.

**Figure 5:**
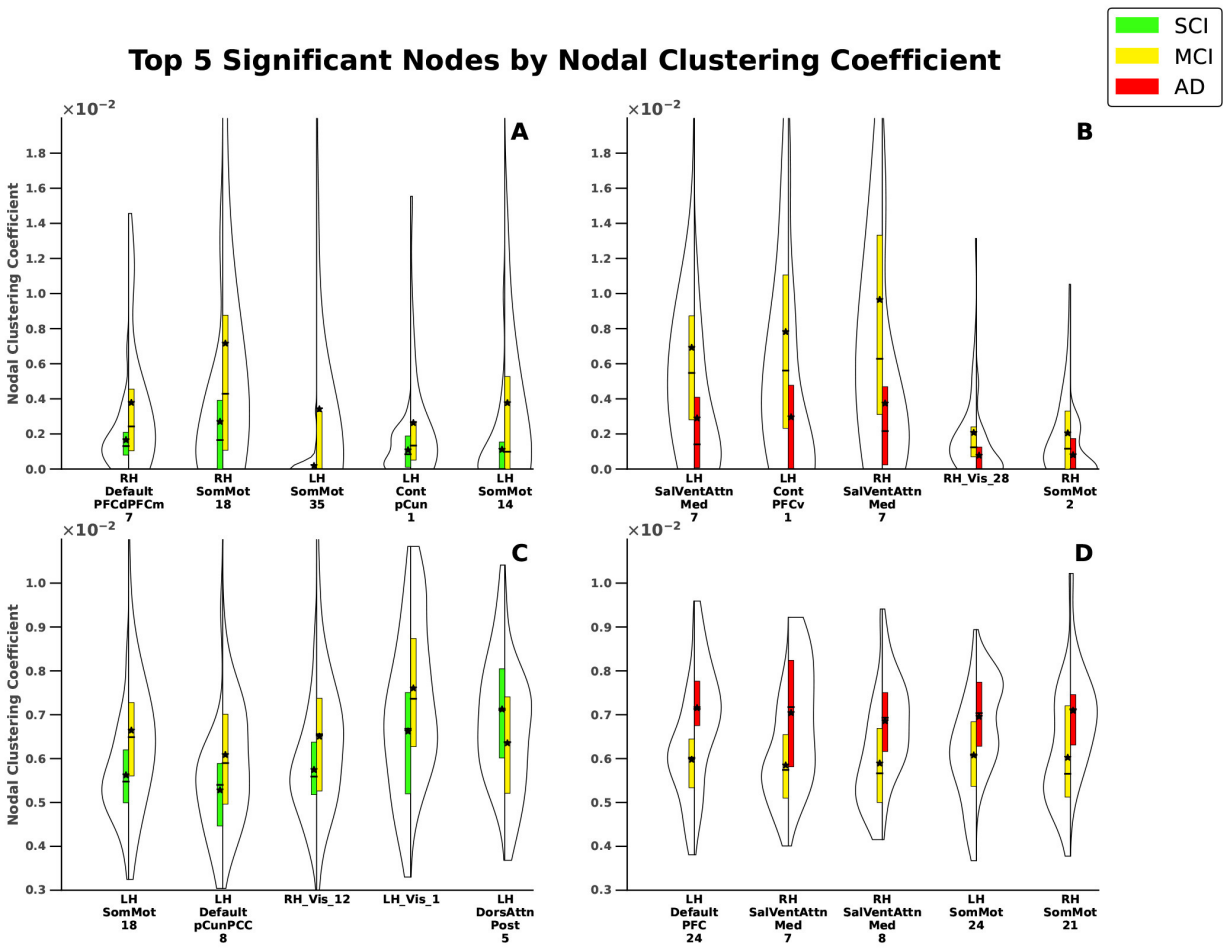
Distributions of nodal clustering coefficient for the top five most discriminative nodes in structural (sNET, top row) and functional (fNET, bottom row) networks across the cognitive decline spectrum. **(A)** Top five most discriminative sNET nodes for the SCI vs. MCI comparison (*p* < 0.011). A statistically significant increase in nodal clustering coefficient is observed in the transition from SCI (green) to MCI (yellow). **(B)** Top five most discriminative sNET nodes for the MCI vs. ADD comparison (*p* < 0.009). In contrast to the early stage, a statistically significant decrease is observed as the disease progresses from MCI (yellow) to ADD (red). **(C)** Top five most discriminative fNET nodes for the SCI vs. MCI comparison (*p* < 0.031). A statistically significant increase in nodal clustering coefficient is observed in the transition from SCI (green) to MCI (yellow). **(D)** Top five most discriminative fNET nodes for the MCI vs. ADD comparison (*p* < 0.003). Unlike the sNET, a continued statistically significant increase is observed from MCI (yellow) to ADD (red). (*) and (-) inside colored bars represent the mean and median of the distributions, respectively.

These trends were further clarified by examining the top five most discriminative nodes for each comparison. For sNETs, a statistically significant increase in the nodal clustering coefficient was observed as cognitive decline progressed from SCI to MCI (*p* < 0.011), as shown in Figure 5A. This upward trend reversed during the transition from MCI to ADD, where a significant decrease was noted (*p* < 0.009), illustrated in Figure 5B. For fNETs, the clustering coefficient of the most discriminative nodes also increased significantly from SCI to MCI (*p* < 0.031), as detailed in Figure 5C. However, unlike in sNETs, these fNET nodes demonstrated a continued statistically significant increase in the transition from MCI to ADD (*p* < 0.003), shown in Figure 5D.

### 3.4 Nodal betweenness centrality

Betweenness centrality (Equation 6), identifying nodes that act as bridges for information flow, was analyzed to identify alterations in global communication pathways across stages of cognitive decline. Figures 2M–P presents the differential nodal betweenness centrality maps derived from Welch's *t*-test, highlighting cortical regions with statistically significant changes. In the SCI-vs.-MCI comparison, 16 significant nodes (*p* < 0.05) were identified among the 400 nodes in the sNETs (Figure 2M), whereas 19 significant nodes were detected in the fNETs (Figure 2O). In the MCI-vs.-ADD comparison, the number of significant nodes increased to 39 in the sNETs (Figure 2N), whereas 12 significant nodes were identified in the fNETs (Figure 2P) (*p* < 0.05).

The maps reveal a stage-dependent and modality-specific pattern. In the sNET, the SCI-vs.-MCI comparison was characterized predominantly by increased betweenness centrality, whereas the MCI-vs.-ADD comparison was dominated by decreases, suggesting a transition from early structural compensation to later network breakdown. In the fNET, the SCI-vs.-MCI comparison showed a mixed pattern, with 12 nodes showing increased and seven nodes showing decreased betweenness centrality. In contrast, the MCI-vs.-ADD comparison was dominated by decreases, with only three nodes showing increased and 9 nodes showing decreased betweenness centrality, indicating reduced functional integration in later disease stages.

**Figure 6:**
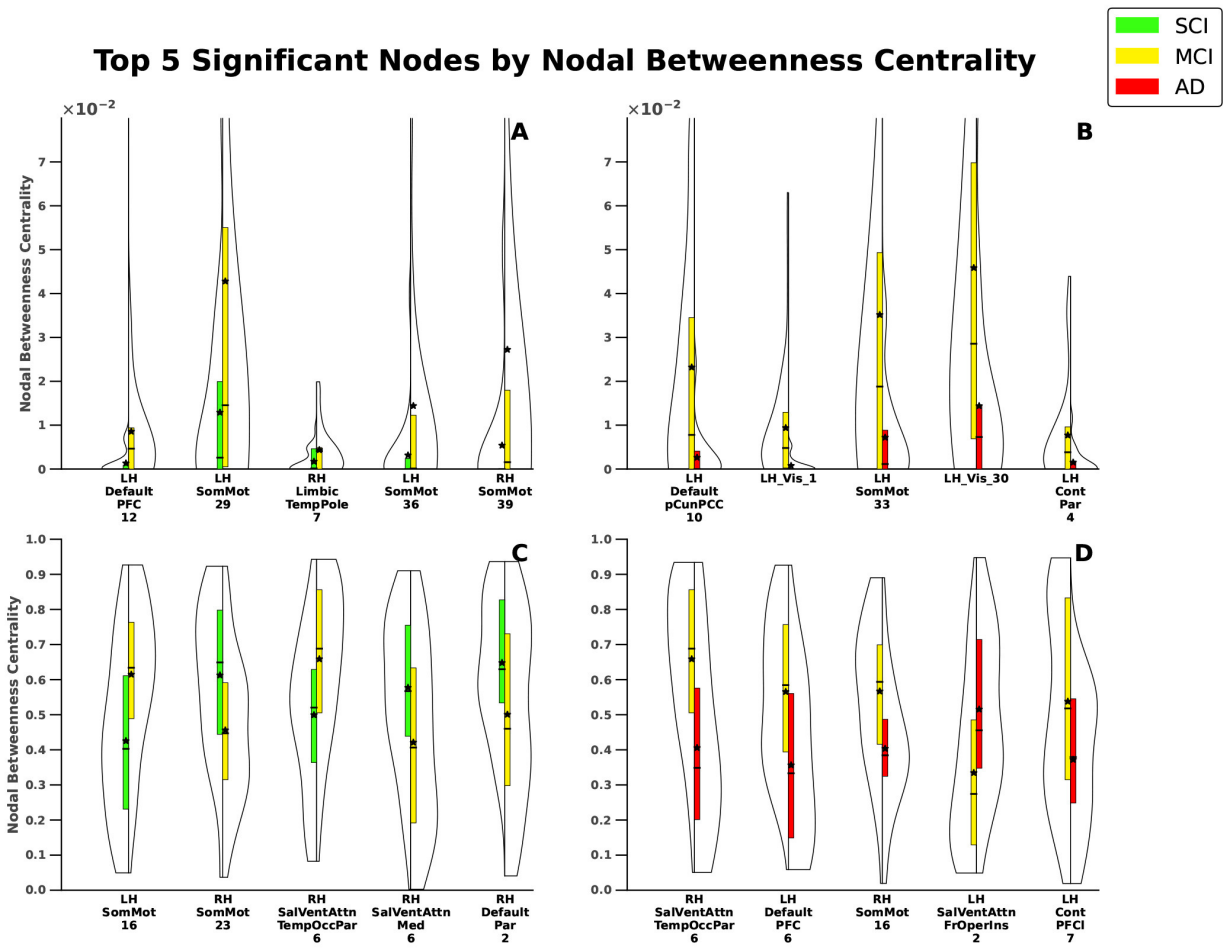
Distributions of nodal betweenness centrality for the top five most discriminative nodes in structural (sNET, top row) and functional (fNET, bottom row) networks across the cognitive decline spectrum. **(A)** Top five most discriminative sNET nodes for the SCI vs. MCI comparison (*p* < 0.021). A statistically significant increase in nodal betweenness centrality is observed in the transition from SCI (green) to MCI (yellow). **(B)** Top five most discriminative sNET nodes for the MCI vs. ADD comparison (*p* < 0.002). A statistically significant decrease is observed as the disease progresses from MCI (yellow) to ADD (red). **(C)** Top five most discriminative fNET nodes for the SCI vs. MCI comparison (*p* < 0.001). No consistent directional change in nodal betweenness centrality is observed across the most discriminative nodes during the SCI to MCI transition. **(D)** Top five most discriminative fNET nodes for the MCI vs. ADD comparison (*p* < 0.011). A statistically significant decrease is observed from MCI (yellow) to ADD (red), with the exception of LH SalVentAttn FrOperIns 2. (*) and (-) inside colored bars represent the mean and median of the distributions, respectively.

In the transition from SCI to MCI, a significant increase in nodal betweenness centrality was observed for the top five most discriminative sNET nodes (*p* < 0.021), as detailed in Figure 6A. The fNET analysis for this stage did not reveal a consistent directional change across the most discriminative nodes (*p* < 0.009), as shown in Figure 6C. Conversely, during the progression from MCI to ADD, the analysis of sNETs demonstrated a statistically significant decrease in betweenness centrality for the top five discriminating nodes (*p* < 0.002), which is illustrated in Figure 6B. In the fNETs, a similar trend of decreasing centrality was observed for the MCI-vs.-ADD classification (*p* < 0.011). Notably, one node, the left salience/ventral attention frontal operculum insula, showed an exception, as depicted in Figure 6D.

### 3.5 Nodal network measures as candidate diagnostic biomarkers

To evaluate the relevance of the selected nodes as low-dimensional candidate diagnostic biomarkers, we trained linear Support Vector Machine (SVM) classifiers. These were tested separately for each pairwise group comparison (SCI-vs.-MCI, MCI-vs.-ADD, and, for comparison, SCI-vs.-ADD) and for each connectome type (structural and functional) using a leave-one-out cross-validation methodology.

The SCI-vs.-ADD comparison was included solely as a methodological benchmark rather than a clinically actionable classification setting. Since SCI and ADD represent the two extremes of the clinical spectrum examined in this study, this comparison provides an upper bound on the discriminative power achievable from the nodal metrics under the maximum degree of clinical separation. It is intended to aid the interpretation of the relative performance of the SCI-vs.-MCI and MCI-vs.-ADD classifiers, since early detection in practice requires discrimination between adjacent clinical stages.

The experiments were designed to evaluate the classification performance of low-dimensional feature vectors derived from the top five most discriminative nodes (5D) against those based on the full connectome (400-D) as a high-dimensional baseline (Schouten et al., 2017), across four nodal features: clustering coefficient (CC), betweenness centrality (BC), nodal strength (NS), and nodal degree (ND).

Table 2 summarizes the classification accuracies obtained across all evaluated settings. Overall, the 5D feature vectors achieved competitive or superior classification performance relative to the full 400-D connectome representation across pairwise group comparisons, connectome types, and nodal measures, while offering substantially greater interpretability. Among the structural connectome models, the highest accuracy was observed for betweenness centrality in the SCI-vs.-ADD benchmark comparison (0.82), whereas nodal degree also showed relatively strong performance for SCI-vs.-ADD (0.78). In the functional connectome, the best accuracies were obtained for clustering coefficient in the MCI-vs.-ADD comparison (0.76) and for betweenness centrality in the SCI-vs.-MCI and SCI-vs.-ADD comparisons (0.78 for both).

**Table 2:** Classification performance across comparison and nodal graph metrics^*^.

Network	Comparison	BC	CC	NS	ND
sNET	SCI–MCI	0.73/0.55*/0.51^†^	0.77/0.60*/0.52^†^	0.74/0.53*/0.43^†^	0.70/0.56*/ 0.52^†^
	MCI–ADD	0.74/0.60*/0.48^†^	0.66/0.53*/0.51^†^	0.66/0.55*/ 0.50^†^	0.72/0.64*/0.57^†^
	SCI–ADD	0.82/0.52*/0.56^†^	0.75/0.52*/0.48^†^	0.75/0.68*/0.57^†^	0.78 / 0.75*/ 0.55^†^
fNET	SCI–MCI	0.78/0.58*/0.52^†^	0.78/0.48*/0.51^†^	0.70/0.50*/0.51^†^	0.68/0.53*/ 0.53^†^
	MCI–ADD	0.66/0.43*/0.49^†^	0.65/0.62*/0.55^†^	0.76/0.57*/0.58^†^	0.70/0.52*/ 0.58^†^
	SCI–ADD	0.78/0.45*/0.42^†^	0.72/0.65*/0.56^†^	0.77/0.50*/0.52^†^	0.72/0.53*/ 0.53^†^

Values are reported as 5D accuracy/400-D accuracy/permutation-null accuracy. All true accuracies (5D) significantly exceeded the empirical null distribution (*p* < 0.05, 1,000 iterations). BC, betweenness centrality; CC, clustering coefficient; NS, nodal strength; ND, nodal degree. ^†^Permutation-based null accuracy.

Further, a permutation-based null model was evaluated as a baseline comparison. The corresponding null accuracies generally remained near chance level, providing supportive evidence that the observed classification performance was not solely driven by random label structure. All classification accuracies, including the 5D and 400-D comparisons and the permutation-based null accuracies, are reported in Table 2.

To complement the raw accuracy values reported in Table 2, balanced accuracy, sensitivity, specificity, and AUC, each accompanied by 95% Wilson confidence intervals, are provided in Supplementary Tables S12–S16. These class-imbalance-robust metrics are broadly consistent with the raw accuracy patterns and confirm that the reported performance is not primarily driven by exploiting the unequal group sizes. Comparable performance was also observed for an L2-regularized logistic regression baseline trained under identical LOOCV and within-fold feature selection (Supplementary Table S17), supporting the interpretation that the discriminative signal resides in the selected nodal features rather than in classifier-specific properties.

To quantify the magnitude and precision of group differences at the node level, Hedges' *g* effect sizes and 95% confidence intervals for the mean differences of the top five discriminative nodes are reported for all comparisons, networks, and metrics in Supplementary Tables S7–S10.

Additionally, to assess whether these findings were influenced by age, we conducted two complementary analyses. First, as a *post-hoc* screening step, Pearson's correlation coefficients and linear regression models were computed between age and each of the four nodal measures across the identified nodes. All absolute correlation coefficients remained below 0.28, *R*^2^ values below 0.06, and no F-test reached significance at α = 0.05. Second, as a confound-adjusted sensitivity analysis, age was incorporated directly as an additional sixth feature alongside the top-5 nodal features within the LOOCV-SVM framework. Including age did not produce a consistent improvement in classification accuracy across comparisons or network types, and in several cases accuracy decreased relative to the 5-feature models. Together, both analyses suggest that the predictive utility of the identified nodal features is not primarily driven by age; detailed results are provided in Supplementary Tables S2–S6.

## 4 Discussion

In this study, we systematically examined local network changes in brain connectivity across the spectrum of cognitive decline, providing a comprehensive overview of how nodal measures in structural and functional networks evolve through the course of Alzheimer's disease. We note that, in the absence of a cognitively normal control group, all reported stage-transition patterns should be interpreted relative to the SCI clinical group, which itself may already exhibit subtle neurobiological alterations compared with healthy aging. Our analysis of nodal degree, strength, clustering coefficient, and betweenness centrality suggests a coherent stage-dependent pattern, findings consistent with early compensatory-type reorganization that may give way to progressive structural decline alongside persistent functional reorganization.

The early transition from SCI to MCI appears to be associated with a coordinated pattern of network reorganization across multiple nodal graph measures. We observed a significant increase in nodal degree and strength in both sNETs and fNETs, consistent with possible network reorganization in which the brain may strengthen existing connections or recruit additional pathways. This pattern may reflect a demand-driven response to early neuronal changes, although causal inference is not possible in a cross-sectional study. Concurrently, the increase in the clustering coefficient in both network types suggests that this reinforcement is highly organized at a local level, forming tighter and more redundant computational communities to preserve local processing efficiency. At a global level, the increase in betweenness centrality, particularly in the sNET, points to an adaptive reorganization where specific pathways become more critical, efficiently rerouting information through emerging hub regions. This convergence across structural and functional metrics is consistent with an early system-level compensatory-type response, although longitudinal data would be needed to strengthen this interpretation.

However, as the disease advances from MCI to ADD, this pattern appears to weaken, with structural and functional networks showing greater divergence. The sNET undergoes a comprehensive and progressive degradation. We observed a marked decrease in sNET degree and strength, consistent with a breakdown of structural network infrastructure and potentially reflecting cumulative axonal and synaptic changes. This loss of connections is accompanied by a decrease in the clustering coefficient, indicating the disintegration of local network cohesion, and a substantial drop in betweenness centrality, reflecting the deterioration of previously crucial hub nodes and white matter pathways. These combined changes are consistent with progressive fragmentation of the structural network, with implications for both local integrity and global resilience.

In stark contrast, the fNET exhibits a more complex and persistent, albeit ultimately insufficient, adaptive response during the MCI-to-ADD transition. Functional degree, strength, and clustering continue to increase, suggesting a sustained pattern that may reflect compensatory-type reorganization in the presence of accumulating structural damage, though this interpretation is necessarily speculative in the absence of longitudinal data and direct measures of cognitive reserve or synaptic integrity. This may reflect an increased recruitment of alternative neural circuits or heightened synchronicity in remaining pathways, intensifying local communication in an attempt to maintain function. Yet, this localized hyper-connectivity is not enough. The predominant decrease in functional betweenness centrality during this same period is a critical finding, as it signifies that the network's capacity for efficient, global communication is severely constrained. The functional network, while perhaps locally overactive, appears less capable of maintaining global integration. This imbalance, in which local reorganization may be insufficient to preserve global network efficiency, is consistent with a tipping point at which functional decline becomes predominant, although this interpretation would benefit from prospective longitudinal validation.

The classification results in Table 2 reveal that the low-dimensional (5D) feature vectors derived from the top five discriminative nodes achieve performance competitive with, and in several cases exceeding, the full 400-D connectome representation. This provides a key insight: the most relevant diagnostic information for Alzheimer's progression is concentrated within a sparse subset of critical brain regions, rendering high-dimensional representations more redundant and less interpretable. These findings support a targeted feature-selection strategy, although external validation in independent cohorts is required before any clinical translation.

Several limitations should be acknowledged. First, this study employed an exploratory statistical framework that prioritized sensitivity over specificity in detecting candidate nodal biomarkers. We deliberately did not apply family-wise error rate (FWER) correction, instead relying on a two-stage validation approach: liberal univariate screening (*p* < 0.05, uncorrected) followed by rigorous permutation-tested classification (1,000 iterations). While this framework demonstrates that our top-5 node sets support above-chance prediction, confirming genuine disease-related signals (Table 2), we acknowledge that not all individually significant nodes may replicate in independent cohorts. The spatially coherent reorganization patterns (Figure 2) and cross-modal convergence between sNET and fNET strengthen confidence in the biological validity of our findings, but external validation remains essential to distinguish stable biomarkers from dataset-specific fluctuations.

Second, the sample size was modest and diagnostically imbalanced, particularly for the ADD group (n = 18). Under leave-one-out cross-validation, small and imbalanced groups increase sensitivity to individual outliers, which may inflate or deflate accuracy estimates for the MCI-vs.-ADD comparison. To partially mitigate this, we verified that classification performance remained above the permutation-based null distribution (*p* < 0.05) for all reported comparisons, providing a leakage-free lower bound on discriminative signal. Nonetheless, future studies with larger, balanced cohorts are needed to confirm these patterns and to improve detection of subtle early-stage alterations, particularly in the SCI-to-MCI transition.

Third, atlas selection is a consequential modeling decision in graph-theoretic connectome analysis, as parcel boundaries directly shape nodal features and downstream graph statistics (Phillips et al., 2015). The Schaefer-400 atlas was chosen because it jointly optimizes local functional homogeneity and global network structure across 1489 participants, yielding parcels that outperform prior atlases on connectional homogeneity metrics while preserving the Yeo seven-network organization (Schaefer et al., 2017). The 400-parcel resolution also approximates the estimated number of functionally distinct cortical areas in the human brain, thereby balancing anatomical specificity with connectivity-estimation stability. The Yeo-7 network labels were used here strictly as a coarse *post-hoc* naming framework for contextualizing significant nodes; no statistical tests were conducted at the Yeo-7 network level. We note that interpreting highly localized parcel-level findings through a seven-network taxonomy risks overstating precision, and that registration accuracy, subject-level spatial variability, and small-region BOLD signal stability all become relevant when fine-grained parcels drive the main results (Phillips et al., 2015; Du et al., 2020). Replication under alternative parcellation schemes will be needed to establish the atlas-independence of the reported nodal effects.

Fourth, although our classification analyses yielded encouraging performance across multiple metrics and network types (Table 2), these results require validation on independent datasets before clinical translation. Prospective studies should also incorporate longitudinal designs to establish whether the observed nodal alterations predict individual-level disease progression, moving beyond cross-sectional group comparisons to personalized prognostic models.

Fifth, the rs-fMRI acquisition (TR = 3,000 ms, 200 volumes, ~10 min) was aligned with the ADNI basic resting-state protocol, which supports comparability with the broader AD-spectrum literature. The 200-volume acquisition is also consistent with current recommendations for elastic-net partial correlation estimation (Lim et al., 2019), and registration quality was visually verified by experienced neurologists for all participants. We nevertheless acknowledge that formal test–retest reliability of the functional graph measures was not assessed. In the supplementary analyses, split-half edge-level stability was low (median *r* = 0.215), falling below thresholds typically considered acceptable for individual-level functional connectivity estimation. The sliding-window analysis yielded more moderate consistency, though this metric is partially inflated by the high temporal overlap between windows. These findings indicate that subject-level fNET estimates remain sensitive to acquisition length and preprocessing choices, and should be interpreted with appropriate caution.

Additionally, the primary fMRI preprocessing pipeline did not include global signal regression, WM/CSF nuisance regression, scrubbing/censoring, or archived motion summary measures such as framewise displacement or DVARS. To evaluate sensitivity to global signal handling, we performed an additional parcel-level GSR sensitivity analysis. This showed that the overall functional-connectivity structure remained broadly preserved under GSR, but the proportion of negative edges and network density changed modestly across groups, indicating that some signed graph properties remain sensitive to GSR (Fox et al., 2005; Liu et al., 2017; Li et al., 2019; Ciric et al., 2017). Supplementary full-sample No-GSR vs. GSR comparisons further suggested that top-ranked discriminative nodes and apparent classification behavior were not fully stable across preprocessing choices. These supplementary results are descriptive rather than leakage-controlled predictive estimates. Taken together, the absence of WM/CSF nuisance regression, motion censoring, and archived motion quality-control metrics, combined with the limited split-half edge-level stability, means that the fNET findings reported here should be treated as exploratory and preprocessing-sensitive. Future studies with full confound pipelines will be necessary to establish the robustness of the functional connectome results.

We note several indirect considerations that argue against a purely motion-driven explanation for the reported functional findings: the convergence of directional patterns across sNETs (derived from diffusion tractography) and fNETs, the reversal in group-difference direction between the SCI–MCI and MCI–ADD comparisons, and the anatomical concentration of discriminative nodes within default-mode, limbic, and salience/ventral-attention systems known to be selectively vulnerable in Alzheimer's disease (Braak and Braak, 1991; Seeley et al., 2007; Jones et al., 2016). These arguments are indirect, however, and future studies with archived motion quality-control metrics and explicit nuisance regression will be needed to address this issue more definitively.

The biological relevance of the discriminative nodes can be interpreted in light of their distribution across large-scale brain systems known to be affected in Alzheimer's disease. Nodes involving the precuneus/posterior cingulate and medial prefrontal components of the default mode network are particularly plausible, given prior reports of structural and functional default mode network abnormalities in both MCI and Alzheimer's disease (Zhou et al., 2022; Weiler et al., 2014), as well as evidence for cascading network failure across the Alzheimer's disease spectrum (Jones et al., 2016). Likewise, limbic and temporal/orbitofrontal nodes are biologically meaningful because these regions are implicated early in Alzheimer-related neuropathological staging and have been associated with altered structural and functional topology in prior network studies (Braak and Braak, 1991; Lin et al., 2019; Qi et al., 2019). The salience/ventral attention and insular–frontal opercular nodes may reflect abnormalities in systems involved in salience detection and network switching (Seeley et al., 2007; Menon, 2013), whereas the emergence of somatomotor and visual nodes in later-stage discrimination may indicate broader propagation of network disruption beyond early associative hubs (Albers et al., 2015; Yu et al., 2021).

## 5 Conclusion

In conclusion, this study provides a multimodal characterization of stage-dependent network reorganization across the SCI–MCI–ADD spectrum. The observed patterns suggest a progression from early changes consistent with compensatory-type reorganization to later structural and functional disruption, offering a more nuanced view of how cognitive decline may affect brain network organization. Given the cross-sectional and exploratory nature of the present work, these findings should be interpreted as hypothesis-generating rather than confirmatory. Nonetheless, the identified nodal metrics may represent candidate markers of disease-related network change and merit further evaluation in independent cohorts and longitudinal studies.

These findings are consistent with previous connectome studies showing that Alzheimer's disease involves both early network reorganization and later large-scale disconnection, rather than a simple linear loss of connectivity (Tijms et al., 2013; Yu et al., 2021). In particular, the stage-dependent pattern observed here—characterized by early increases in several nodal measures and later structural decline with reduced integrative capacity—is consistent with the possibility that compensatory-type network changes may precede progressive network breakdown across the SCIMCIADD spectrum, in line with earlier structural and functional network studies in Alzheimer's disease (Xiang et al., 2013; Pereira et al., 2018; Wang et al., 2016). From a pathophysiological perspective, the early increases in nodal degree, strength, clustering coefficient, and, in part, betweenness centrality may reflect compensatory reorganization in response to emerging synaptic dysfunction and reduced network efficiency in vulnerable systems. In contrast, the later decrease observed particularly in structural nodal measures during the MCI-to-ADD transition is more consistent with progressive axonal degeneration, synaptic loss, and disruption of white-matter-mediated communication. At the same time, the persistence of increased functional nodal measures in later stages may indicate recruitment of alternative pathways that is insufficient to preserve communication despite ongoing structural breakdown. The involvement of regions belonging to the default mode, limbic, salience/ventral attention, and somatomotor systems is also concordant with previous reports highlighting the selective vulnerability and reorganization of large-scale brain networks in Alzheimer's disease (Yu et al., 2021; Yuksel Dal et al., 2024). Taken together, our results extend the current literature by showing that low-dimensional nodal markers derived from multimodal connectomes may provide an interpretable framework for tracking stage-dependent brain reorganization in cognitive decline.

## Data Availability

The datasets presented in this article are not readily available as data sharing requires approval from the funding agency (TUBITAK). Requests to access the datasets should be directed to demetyukseldal@gmail.com.

## Footnotes

1 Istanbul University, Istanbul Faculty of Medicine, Ethics Committee Approval: 877/30.05.2014.

2 Boğaziçi University, Ethics Committee Approval: 2014-1/17.02.2014.

3 The *DiffusionTensor3DReconstructionImageFilter* from ITK v6.0.0 is used.
