# Supplementary material for: Local brain connectome parameters across the spectrum of clinical cognitive decline
Source: Front Neurosci. 2026 Jun 19;20:1840382. doi: 10.3389/fnins.2026.1840382 (PMC13328281; doi:10.3389/fnins.2026.1840382)
Supplement: Supplementary file 1 [file Data_Sheet_1.pdf]

Supplementary Material

1 DATA PREPROCESSING

The preprocessing pipeline integrates structural (T1w, T2w, DWI) and functional (fMRI) MRI data, together with Schaefer parcellation and WM/GM/CSF segmentation masks, into MNI152 space at $1\,mm$ isotropic resolution (Fonov et al., 2011). The workflow is implemented as an automated in-house pipeline (Fig. S1) that combines established tools: FSL (fsl, 2019) for registration and fMRI cleanup, FreeSurfer (fre, 2019) for cortical surface reconstruction and parcellation, and TORTOISE (tor, 2019) (DIFFPREP/DIFFCALC) for diffusion preprocessing. The overall procedure first minimizes motion and scanner artefacts (motion correction, distortion correction, bias field correction) and produces co-registered, skull-stripped T1w/T2w images, parcellations, and tissue masks suitable for multimodal one-to-one network mapping.

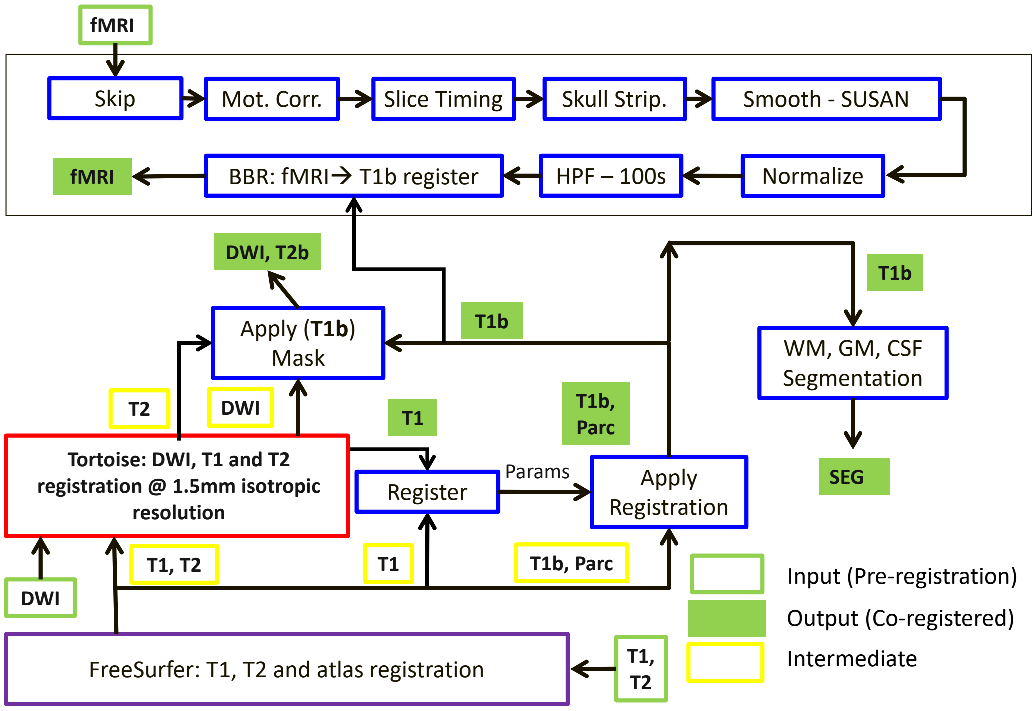

Figure S1: Preprocessing Pipeline Diagram. The utilization of the Tortoise, FreeSurfer, and FSL programs is depicted by red, purple, and blue boxes, respectively. Parc refers to the Schaefer-400 Atlas parcellation.

Structural preprocessing follows the HCP pre-FreeSurfer and FreeSurfer stages (Glasser et al., 2013; hcp, 2019) (see Figs. S2–S3). In pre-FreeSurfer, original T1w/T2w images undergo AC–PC alignment, brain extraction, transformation to native volume space, and distortion correction; Boundary-Based Registration (BBR) is then used to refine cross-modal alignment (Greve and Fischl,

2009), and bias-field correction equalizes intensity nonuniformities before exporting native-space, skull-stripped volumes. These outputs feed FreeSurfer's `recon-all` (autorecon1/2) to reconstruct white-matter and pial surfaces, perform gray-matter intensity normalization, remove dura/vessels guided by T2w information, and register surfaces to the standard template, while producing the Schaefer atlas parcellation and detailed subcortical segmentations (rec, 2024).

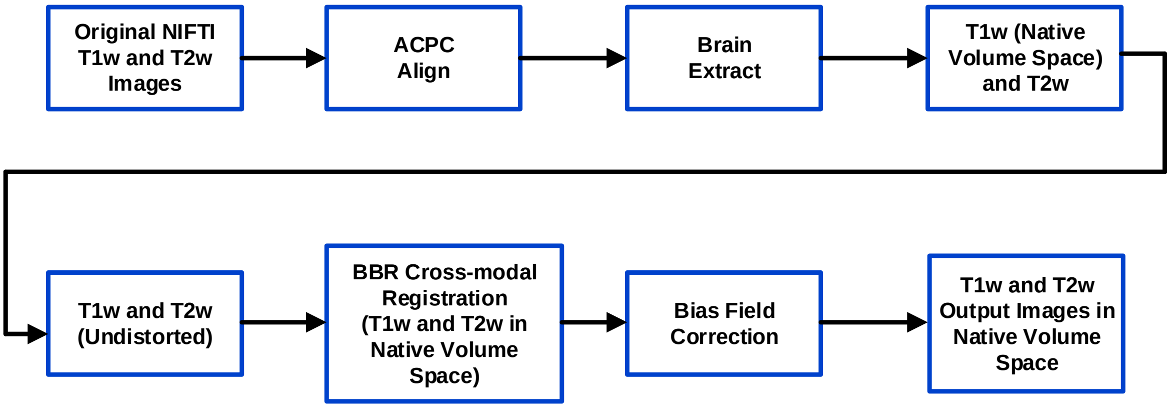

Figure S2: Pre-FreeSurfer Pipeline Block Diagram

Diffusion data are processed with TORTOISE's DIFFPREP/DIFFCALC modules to correct motion and eddy/distortion effects, reorient and resample volumes, and perform error analysis; final DWI (and associated T1w/T2w derivatives used for diffusion registration) are produced at 1.5 mm resolution. Registration parameters are estimated in FSL using the high-resolution FreeSurfer T1w (1 mm) and the TORTOISE T1w (1.5 mm) and then applied to the skull-stripped T1b and Schaefer parcellation. The refined T1b is the reference for tissue segmentation (WM/GM/CSF), for generating skull-stripped T2b, and for aligning DWI outputs, ensuring consistent anatomical anchors across modalities.

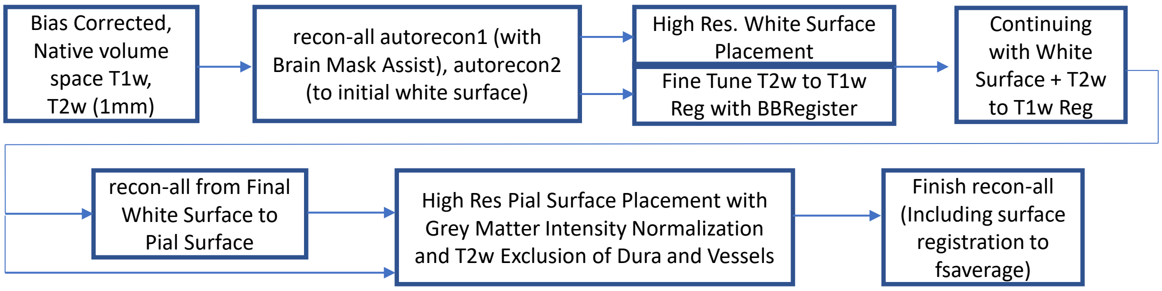

Figure S3: FreeSurfer pipeline block diagram

fMRI preprocessing (FSL) removes the first five volumes, applies motion correction and slice-timing correction, and performs skull stripping and spatial smoothing (SUSAN) (Smith and Brady, 1997). A temporal high-pass filter (0.01 Hz cutoff) reduces low-frequency drift, and the denoised functional data are registered to the subject T1w using BBR to achieve precise structure–function alignment. Together, these steps yield spatially harmonized, artifact-reduced datasets that support reproducible multimodal connectome construction and subsequent statistical analyses.

2 CONNECTOME CONSTRUCTION DETAILS

2.1 Structural Network (sNET) Thresholding

Following edge weight computation using the volume-normalised streamline density formula (Equation 1 in the main manuscript), all edges with a normalised weight below 0.01 were set to zero. This threshold serves as a noise floor to remove spurious low-weight connections that are likely attributable to tractography false positives rather than genuine white matter pathways. An absolute threshold of 0.01 on normalised streamline density is consistent with established practice in structural connectomics: (Coombes et al., 2018) demonstrated that a connectivity threshold at this level excludes connections with high between-subject variability and poor reproducibility, and recommended thresholds ≥ 0.01 for stable graph-metric estimation. Following thresholding, the resulting sNET adjacency matrices are sparse and weighted; degree and clustering coefficient are computed on the binarised form of these matrices, while strength retains the original normalised weights, as described in Section 2.3 of the main manuscript.

2.2 Functional Network (fNET) Construction Parameters

Functional connectivity was estimated via elastic-net regularised partial correlation using MATLAB's `lasso` function with the elastic-net penalty option. The elastic-net combines an ℓ_1 (LASSO) penalty, which promotes sparsity, with an ℓ_2 (Ridge) penalty, which stabilises coefficient estimates under multicollinearity, a known property of parcel-wise BOLD signals in densely connected cortical atlases.

2.2.1 Alpha grid.

The mixing parameter α, which controls the relative weight of the ℓ_1 and ℓ_2 penalties ($\alpha = 1$: pure LASSO; $\alpha = 0$: pure Ridge), was optimised across a grid of 20 values: $\alpha \in \{0.02, 0.07, 0.12, \ldots, 0.97\}$ (step ≈ 0.05), spanning near-Ridge to near-LASSO regularisation.

2.2.2 Lambda selection.

For each parcel and each value of α, the regularisation parameter λ was selected via 10-fold cross-validation using minimum mean squared error (MSE) as the tuning criterion. Optimal α and λ were therefore selected jointly per parcel, yielding a 400-element vector of optimal parameter pairs per subject.

2.2.3 Standardisation.

Parcel-wise BOLD summary signals were standardised (zero mean, unit variance) prior to elastic-net fitting. This ensures that regularisation penalties are applied on a comparable scale across parcels with different signal amplitudes.

2.2.4 Handling of negative and weak edges.

Partial correlation values derived from the elastic-net coefficients may be negative, reflecting inhibitory or competitive relationships between parcels. Negative edges were retained in the fNET without modification. The minimum observed partial correlation across all subjects and parcel pairs was -0.337, confirming that a non-trivial proportion of edges carry negative weights. Since strength is defined as the sum of all edge weights (Equation 3 in the main manuscript) and is computed on the signed weighted matrix, negative edges reduce nodal strength. Degree and clustering coefficient, which depend on the binarised adjacency matrix, are computed after setting all non-positive edges to zero, such that only positive partial correlations contribute to the binary graph structure. Mean network density across subjects was 0.147 ± 0.034 (median density across the two half-scan splits: 0.080 and 0.078 respectively).

2.3 Global Signal Regression Sensitivity Analysis

To assess the sensitivity of the functional-connectivity findings to global signal handling, we performed an additional parcel-level GSR sensitivity analysis across the AD, MCI, and Healthy groups. Starting from the same parcel-wise BOLD summary signals used in the primary pipeline, a global signal was computed as the mean signal across all Schaefer-400 parcels at each time point and regressed out from each parcel time series. Functional networks were then reconstructed using the same subject-specific elastic-net parameters selected in the primary analysis, without re-optimisation, in order to isolate the effect of global signal handling from repeated hyperparameter selection.

Table S1. GSR sensitivity summary for Schaefer-400 functional connectomes.

Group	N	Edge Corr.	IQR	Strength Corr.	IQR	Neg.*	IQR	Neg.†	IQR	Den.*	IQR	Den.†	IQR
AD	18	0.915	0.018	0.968	0.037	0.229	0.026	0.252	0.023	0.468	0.051	0.473	0.049
MCI	46	0.927	0.020	0.963	0.036	0.227	0.015	0.252	0.015	0.463	0.027	0.473	0.028
Healthy	24	0.925	0.037	0.963	0.037	0.226	0.010	0.250	0.012	0.460	0.020	0.468	0.024

Abbreviations: Edge Corr., edge correlation between No-GSR and GSR networks; Strength Corr., nodal strength correlation between No-GSR and GSR networks; Neg., negative edge ratio; Den., network density; * No-GSR values; † GSR values; IQR, interquartile range.

Across all three groups, No-GSR and GSR functional networks remained highly correlated at the edge level (median Spearman $r = 0.915$ in AD, 0.927 in MCI, and 0.925 in Healthy) and at the nodal-strength level (median Spearman $r = 0.968$ in AD, 0.963 in MCI, and 0.963 in Healthy), indicating that the overall functional-connectivity structure was broadly preserved under GSR. At the same time, GSR increased the proportion of negative edges (AD: $0.229 \rightarrow 0.252$; MCI: $0.227 \rightarrow 0.252$; Healthy: $0.226 \rightarrow 0.250$) and slightly increased network density (AD:

$0.468 \rightarrow 0.473$; MCI: $0.463 \rightarrow 0.473$; Healthy: $0.460 \rightarrow 0.468$), consistent with known effects of GSR on signed connectivity structure.

These findings suggest that the reported fNET results are not entirely driven by the absence of GSR, while also confirming that some graph properties remain sensitive to global signal handling. Because this was implemented as a parcel-level sensitivity analysis rather than a full voxelwise reprocessing pipeline, the results should be interpreted as supportive rather than definitive.

2.4 Functional Network Stability

To assess the robustness of the subject-level fNET estimates, two stability analyses were conducted. First, a split-half analysis was performed by dividing each subject's 200-volume time series into two non-overlapping halves and computing the fNET independently for each half. The Pearson correlation between corresponding edge weights across the two half-scan fNETs was used as a measure of within-subject stability. Across subjects, the median split-half edge correlation was $r = 0.215$ (IQR $= 0.039$), and the median split-half strength correlation was $r = 0.376$ (IQR $= 0.149$). Second, a sliding-window analysis was performed using 10 windows of 80% of the total scan duration (`windowFrac` $= 0.80$), with the median Pearson correlation between windowed and full-scan edge weights used to quantify temporal stability. The median window-based edge stability was $r = 0.572$ (IQR $= 0.037$), and the median window-based strength stability was $r = 0.716$ (IQR $= 0.080$). These analyses indicate limited split-half stability, particularly at the edge level, and more moderate consistency under the sliding-window analysis. Together with the GSR sensitivity results, they suggest that the overall functional-connectivity structure is not wholly unstable, but that subject-level fNET measures remain sensitive to acquisition length and preprocessing choices.

3 AGE CORRELATION

To assess whether age may act as a confounding factor in the interpretation of our network-based findings, we examined the correlation between age and four key nodal metrics: nodal degree, nodal strength, clustering coefficient, and betweenness centrality. Analyses were conducted separately for both the sNETs and fNETs across the clinical spectrum from SCI to MCI and from MCI to ADD.

Pearson's correlation coefficients (r) were calculated between age (independent variable) and each of the four nodal metrics (dependent variables) at the identified nodes (see Tables S2, S3, S4, S5). The absolute values of r ranged from 0.007 to 0.28, indicating negligible to weak linear relationships (i.e., the strength and direction of the linear association). For each node we also fitted a linear regression model to evaluate the predictive contribution of age: the coefficient of determination (R^2) ranged from 0.0001 to 0.06, meaning the models explained approximately 0.01% to 6% of the variance in the nodal metrics. Finally, the F-test p-values for the regression models ranged from 0.08 to 0.93 (all above the conventional $\alpha = 0.05$), indicating the effects of age were not statistically significant. These results, small $|r|$, low R^2, and non-significant p-values, consistently demonstrate that none of the examined nodal degree, nodal strength, clustering coefficient, or betweenness centrality values showed statistically significant correlations with age. This suggests that the patterns observed in these nodal metrics are likely driven by disease-related processes rather than by age-related effects.

Table S2. Linear regression and Pearson's correlation analysis for the relationship between age and nodal degree in sNET and fNET, comparing SCI-MCI and MCI-ADD.

		**Node name**	R^2	**p-value**	**r**
**sNET**	SCI–MCI	RH Somatomotor 16	0.0020	0.7135	-0.0447
		LH Limbic Temporal Pole 6	0.020	0.238	0.142
		LH Default Prefrontal Cortex 13	0.026	0.186	0.160
		RH Somatomotor 34	0.008	0.457	0.090
		RH Visual 19	0.013	0.349	0.114
	MCI–ADD	LH Visual 1	0.027	0.196	-0.164
		RH Visual 28	0.023	0.233	0.151
		LH Dorsal Attention Posterior 3	0.002	0.730	0.044
		LH Visual 6	<0.001	0.816	-0.030
		RH Visual 26	<0.001	0.880	-0.019
**fNET**	SCI–MCI	LH Somatomotor 13	0.029	0.152	0.172
		LH Somatomotor 17	0.021	0.227	0.145
		RH Control Precuneus 1	0.016	0.282	0.130
		LH Default Temporal 8	0.010	0.391	0.104
		LH Visual 6	0.006	0.508	0.080
	MCI–ADD	LH Visual 10	0.022	0.241	0.148
		RH default dorsal prefrontal cortex, medial prefrontal cortex 13	0.007	0.4937	0.087
		LH Default Visual 11	0.007	0.487	0.088
		RH Default Temporal 2	0.042	0.102	0.206
		RH Salience / Ventral Attention Precentral 1	0.030	0.171	0.173

Table S3. Linear regression and Pearson's correlation analysis for the relationship between age and nodal strength in sNET and fNET, comparing SCI-MCI and MCI-ADD.

		**Node name**	R^2	**p-value**	**r**
**sNET**	SCI–MCI	LH Somatomotor 35	0.019	0.243	0.141
		RH Somatomotor 16	0.002	0.711	0.045
		LH Somatomotor 31	0.053	0.055	0.230
		RH Somatomotor 39	0.046	0.074	0.214
		RH Default Dorsal Prefrontal Cortex Medial Prefrontal Cortex 4	0.023	0.203	0.153
	MCI–ADD	LH Default Precuneus Posterior Cingulate Cortex 10	<0.001	0.952	0.007
		LH Visual 1	0.006	0.548	-0.076
		LH Somatomotor 33	0.001	0.773	0.036
		LH Visual 30	0.026	0.206	-0.160
		LH Control Parietal 4	0.004	0.622	-0.062
**fNET**	SCI–MCI	LH Somatomotor 17	0.029	0.159	0.170
		LH Somatomotor 13	0.035	0.121	0.186
		LH Limbic Orbital Prefrontal Cortex 4	0.043	0.086	0.207
		LH Salience / Ventral Attentional Medial 6	<0.001	0.865	0.020
		LH Somatomotor 11	0.030	0.153	0.172
	MCI–ADD	LH Visual 10	0.005	0.584	0.151
		RH Somatomotor 23	0.010	0.433	0.099
		RH Default Ventral Prefrontal Cortex 3	0.023	0.233	0.285
		LH Somatomotor 23	0.004	0.621	0.062
		RH Control Temporal 1	0.047	0.087	0.215

Table S4. Linear regression and Pearson's correlation analysis for the relationship between age and clustering coefficient in sNET and fNET, comparing SCI-MCI and MCI-ADD.

		**Node name**	R^2	**p-value**	**r**
**sNET**	SCI–MCI	RH Default Dorsal Prefrontal Cortex Medial Prefrontal Cortex 7	0.019	0.243	0.141
		RH Somatomotor 18	0.002	0.711	0.045
		LH Somatomotor 35	0.053	0.055	0.230
		LH Control Precuneus 1	0.046	0.074	0.214
		LH Somatomotor 14	0.023	0.203	0.153
	MCI–ADD	LH Salience / Ventral Attention Medial 7	<0.001	0.951	0.007
		LH Control Ventral Prefrontal Cortex 1	0.005	0.547	-0.076
		RH Salience / Ventral Attention Medial 7	0.001	0.773	0.036
		RH Visual 28	0.025	0.205	-0.160
		RH Somatomotor 2	0.003	0.622	-0.062
**fNET**	SCI–MCI	LH Somatomotor 18	0.018	0.269	0.133
		LH Default Precuneus Posterior Cingulate Cortex 8	0.051	0.061	0.225
		RH Visual 12	0.045	0.078	0.212
		LH Visual 1	<0.001	0.933	0.010
		LH Dorsal Attention Posterior 5	0.021	0.224	0.147
	MCI–ADD	LH Default Prefrontal Cortex 24	0.035	0.136	0.188
		RH Salience / Ventral Attention Medial 7	0.041	0.106	0.203
		RH Salience / Ventral Attention Medial 7	0.060	0.051	0.244
		LH Somatomotor 24	0.023	0.229	0.152
		RH Somatomotor 21	0.054	0.064	0.232

Table S5. Linear regression and Pearson's correlation analysis for the relationship between age and betweenness centrality in sNET and fNET, comparing SCI-MCI and MCI-ADD.

		Node name	R^2	p-value	r
**sNET**	Sci-mci	LH Default Prefrontal Cortex 12	0.052	0.057	0.228
		LH Somatomotor 29	0.001	0.780	0.033
		RH Limbic Temporal Pole 7	0.013	0.336	0.116
		LH Somatomotor 36	0.048	0.069	0.218
		RH Somatomotor 39	0.042	0.086	0.206
	mci-add	LH Default Precuneus Posterior Cingulate Cortex 10	0.025	0.209	-0.159
		LH Visual 1	0.033	0.149	-0.182
		LH Somatomotor 33	0.004	0.604	-0.066
		LH Visual 30	0.010	0.442	-0.098
		LH Control Parietal 4	0.011	0.414	-0.104
**fNET**	Sci-mci	LH Somatomotor 16	0.002	0.683	0.050
		RH Somatomotor 23	0.007	0.505	0.081
		RH Salience / Ventral Attention Temporal Occipital Parietal 6	<0.001	0.881	-0.018
		RH Salience / Ventral Attention Medial 6	0.002	0.754	-0.038
		RH Default Parietal 2	0.001	0.777	0.0342
	mci-add	RH Salience / Ventral Attention Temporal Occipital Parietal 6	0.003	0.676	-0.053
		LH Default Prefrontal COrtex 6	0.017	0.302	-0.131
		RH Somatomotor 16	0.026	0.205	-0.161
		LH Salience / Ventral Attention Frontal Operculum Insula 2	0.009	0.452	0.096
		LH Control Lateral Prefrontal Cortex 7	0.001	0.827	0.028

Age as a Sixth Classification Feature

To complement the post-hoc correlation analyses presented earlier in this section, we conducted a confound-adjusted sensitivity analysis in which age was included as an additional (sixth) feature alongside the top-5 nodal features selected within each LOOCV fold. All other aspects of the pipeline were identical to the primary analysis. Table S6 reports the resulting LOOCV classification accuracies alongside the corresponding 5-feature-only accuracies.

Including age did not produce a systematic improvement in accuracy. For the SCI–MCI comparison, small accuracy increases were observed in the sNET across all metrics; however, for the MCI–ADD comparison, accuracy decreased in several settings (e.g., BC sNET: $0.83 \rightarrow 0.75$; CC fNET: $0.79 \rightarrow 0.63$; ND fNET: $0.81 \rightarrow 0.73$). The mixed pattern is consistent with the known association between age and disease stage: the three groups differ substantially in mean age (SCI: 56.95 ± 7.54; MCI: 62.04 ± 9.78; ADD: 69.83 ± 8.52) because cognitive decline progresses with advancing age. Adding age as an explicit feature therefore does not cleanly separate disease-related from age-related variance; rather, it partially replicates the group-separating signal already captured

by the nodal features themselves. These results support the interpretation that the nodal features reflect disease-related network reorganisation rather than a confound attributable solely to age.

Table S6. Classification accuracy (LOOCV, linear SVM) when age is included as an additional sixth feature alongside the top-5 nodal features. Values in parentheses are the corresponding 5-feature-only accuracies from Table 1 of the main manuscript for direct comparison. The absence of a consistent accuracy increase across comparisons and network types indicates that age does not act as an independent discriminative confounder separable from disease stage.

Network	Comparison	BC	CC	NS	ND
sNET	SCI–MCI	0.76 (0.73)	0.79 (0.76)	0.75 (0.71)	0.69 (0.66)
sNET	MCI–ADD	0.75 (0.83)	0.70 (0.69)	0.71 (0.64)	0.71 (0.72)
fNET	SCI–MCI	0.78 (0.74)	0.73 (0.79)	0.73 (0.69)	0.64 (0.59)
fNET	MCI–ADD	0.69 (0.83)	0.63 (0.79)	0.74 (0.78)	0.73 (0.81)

4 NULL MODEL ACCURACIES

Table S7 reports the classification accuracies obtained under the null model, where diagnostic labels were randomly permuted prior to each of the 1,000 iterations of the complete analysis pipeline. In each iteration, the shuffled labels were used to re-execute the entire pipeline from scratch — including Welch's t-test-based node ranking, top-K feature selection, and LOOCV-based SVM classification — thereby ensuring that the null distribution accounts for the full pipeline, including the feature selection step. Since no true association exists between the permuted labels and the network features, the classifier cannot learn any discriminative patterns, and its output is expected to approximate chance-level performance. As shown in the table, the observed null accuracies consistently converge to values close to 0.50 ± 0.1 across all tasks, network types, and nodal measures, which is fully consistent with the theoretical expectation for binary classification under random label assignment.

Table S7. Mean ± standard deviation values of null-model accuracies for Node Degree (ND), Node Strength (NS), Clustering Coefficient (CC), and Betweenness Centrality (BC) across classification tasks in sNET and fNET.

	Task	ND	NS	CC	BC
sNET	SCI-MCI	0.49 ± 0.09	0.49 ± 0.08	0.50 ± 0.07	0.50 ± 0.09
	MCI-ADD	0.49 ± 0.10	0.50 ± 0.11	0.49 ± 0.09	0.50 ± 0.08
	SCI-ADD	0.48 ± 0.07	0.50 ± 0.09	0.49 ± 0.11	0.49 ± 0.11
fNET	SCI-MCI	0.50 ± 0.08	0.50 ± 0.09	0.49 ± 0.08	0.50 ± 0.07
	MCI-ADD	0.50 ± 0.09	0.50 ± 0.08	0.50 ± 0.07	0.50 ± 0.09
	SCI-ADD	0.49 ± 0.07	0.50 ± 0.09	0.50 ± 0.08	0.50 ± 0.07

5 EFFECT SIZES AND CONFIDENCE INTERVALS FOR TOP-5 DISCRIMINATIVE NODES

Tables S8, S9, S10, and S11 report Hedges' g effect sizes and 95% bootstrapped confidence intervals for the mean group difference of the top-5 discriminative nodes across all pairwise comparisons,

network types, and graph metrics (120 nodes total). Hedges' g is preferred over Cohen's d for small and unequal sample sizes as it applies a correction factor for sample size bias. None of the 95% confidence intervals for the mean difference crossed zero, confirming stable group separation for all reported candidate nodes. While individual nodes do not survive strict FDR correction at 400 nodes (all $q > 0.05$), this reflects the conservative nature of FDR penalties under small samples with spatially correlated tests; the permutation-tested classification framework in Section 2.4.4 serves as the primary inferential gate. Effect sizes are moderate to large throughout (SCI–MCI: $|g| = 0.45$–1.09; MCI–ADD: $|g| = 0.52$–1.21; SCI–ADD: $|g| = 0.73$–1.34). Positive g values indicate higher values in the later-stage group; negative values indicate lower values in the later-stage group.

Table S8. Hedges' g effect sizes and 95% confidence intervals [lower, upper] for the mean group difference of the top-5 discriminative nodes in Betweenness Centrality (BC), across pairwise comparisons and network types. Positive g: higher value in later-stage group. Negative g: lower value in later-stage group.

Network	Comparison	Node	Mean Diff [95% CI]	Hedges' g
sNET	SCI–MCI	LH_Default_PFC_12	−0.007 [−0.012, −0.002]	−0.54
		LH_SomMot_29	−0.030 [−0.051, −0.009]	−0.55
		RH_Limbic_TempPole_7	−0.003 [−0.005, −0.001]	−0.56
		LH_SomMot_36	−0.011 [−0.021, −0.002]	−0.46
		RH_SomMot_39	−0.022 [−0.040, −0.004]	−0.45
	MCI–ADD	LH_Default_pCunPCC_10	0.021 [0.012, 0.029]	0.83
		LH_Vis_1	0.009 [0.005, 0.013]	0.75
		LH_SomMot_33	0.028 [0.013, 0.043]	0.69
		LH_Vis_30	0.031 [0.014, 0.049]	0.74
		LH_Cont_Par_4	0.006 [0.002, 0.010]	0.61
fNET	SCI–MCI	LH_SomMot_16	−0.189 [−0.304, −0.074]	−0.85
		RH_SomMot_23	0.157 [0.047, 0.266]	0.74
		RH_SalVentAttn_TempOccPar_6	−0.159 [−0.272, −0.047]	−0.72
		RH_SalVentAttn_Med_6	0.156 [0.041, 0.271]	0.63
		RH_Default_Par_2	0.147 [0.039, 0.256]	0.61
	MCI–ADD	RH_SalVentAttn_TempOccPar_6	0.253 [0.107, 0.399]	1.08
		LH_Default_PFC_6	0.209 [0.078, 0.340]	0.94
		RH_SomMot_16	0.163 [0.058, 0.269]	0.81
		LH_SalVentAttn_FrOperIns_2	−0.181 [−0.314, −0.048]	−0.76
		LH_Cont_PFCl_7	0.166 [0.041, 0.291]	0.63

Table S9. Hedges' g effect sizes and 95% confidence intervals [lower, upper] for the mean group difference of the top-5 discriminative nodes in Clustering Coefficient (CC), across pairwise comparisons and network types. Positive g: higher value in later-stage group. Negative g: lower value in later-stage group.

Network	Comparison	Node	Mean Diff [95% CI]	Hedges' g
sNET	SCI–MCI	RH_Default_PFCdPFCm_7	−0.002 [−0.003, −0.001]	−0.62
		RH_SomMot_18	−0.004 [−0.007, −0.002]	−0.60
		LH_SomMot_35	−0.003 [−0.006, −0.001]	−0.49
		LH_Cont_pCun_1	−0.002 [−0.003, −0.000]	−0.51
		LH_SomMot_14	−0.003 [−0.005, −0.001]	−0.51
	MCI–ADD	LH_SalVentAttn_Med_7	0.004 [0.001, 0.007]	0.65
		LH_Cont_PFCv_1	0.005 [0.002, 0.008]	0.69
		RH_SalVentAttn_Med_7	0.006 [0.002, 0.010]	0.57
		RH_Vis_28	0.001 [0.000, 0.002]	0.60
		RH_SomMot_2	0.001 [0.000, 0.002]	0.56
fNET	SCI–MCI	LH_SomMot_18	−0.001 [−0.002, −0.000]	−0.54
		LH_Default_pCunPCC_8	−0.001 [−0.001, −0.000]	−0.55
		RH_Vis_12	−0.001 [−0.001, −0.000]	−0.51
		LH_Vis_1	−0.001 [−0.002, −0.000]	−0.58
		LH_DorsAttn_Post_5	0.001 [0.000, 0.001]	0.56
	MCI–ADD	LH_Default_PFC_24	−0.001 [−0.002, −0.000]	−1.02
		RH_SalVentAttn_Med_7	−0.001 [−0.002, −0.000]	−1.08
		RH_SalVentAttn_Med_8	−0.001 [−0.002, −0.000]	−0.85
		LH_SomMot_24	−0.001 [−0.001, −0.000]	−0.79
		RH_SomMot_21	−0.001 [−0.002, −0.000]	−0.86

Table S10. Hedges' g effect sizes and 95% confidence intervals [lower, upper] for the mean group difference of the top-5 discriminative nodes in Nodal Strength (NS), across pairwise comparisons and network types. Positive g: higher value in later-stage group. Negative g: lower value in later-stage group.

Network	Comparison	Node	Mean Diff [95% CI]	Hedges' g
sNET	SCI–MCI	LH_SomMot_35	−0.075 [−0.108, −0.043]	−0.85
		RH_SomMot_16	−0.093 [−0.138, −0.047]	−0.83
		LH_SomMot_31	−0.054 [−0.083, −0.025]	−0.75
		RH_SomMot_39	−0.244 [−0.380, −0.108]	−0.70
		RH_Default_PFCdPFCm_4	−0.147 [−0.233, −0.062]	−0.69
	MCI–ADD	RH_SalVentAttn_Med_7	0.731 [0.381, 1.081]	0.99
		LH_Vis_29	0.134 [0.054, 0.214]	0.62
		RH_Vis_10	0.036 [0.010, 0.062]	0.54
		LH_Vis_1	0.089 [0.025, 0.152]	0.53
		RH_SomMot_30	0.249 [0.069, 0.429]	0.52
fNET	SCI–MCI	LH_SomMot_17	−0.482 [−0.692, −0.272]	−1.09
		LH_SomMot_13	−0.453 [−0.740, −0.167]	−0.80
		LH_Limbic_OFC_4	−0.418 [−0.692, −0.145]	−0.71
		LH_SalVentAttn_Med_6	−0.396 [−0.659, −0.133]	−0.68
		LH_SomMot_11	−0.341 [−0.578, −0.105]	−0.63
	MCI–ADD	LH_Vis_10	−0.574 [−0.901, −0.246]	−1.14
		RH_SomMot_23	−0.469 [−0.788, −0.149]	−0.89
		RH_Default_PFCv_3	−0.483 [−0.831, −0.135]	−0.81
		LH_SomMot_23	−0.428 [−0.740, −0.117]	−0.85
		RH_Cont_Temp_1	−0.526 [−0.913, −0.140]	−0.93

6 EXTENDED CLASSIFICATION PERFORMANCE METRICS

Tables S12–S16 report accuracy, balanced accuracy, sensitivity, specificity, and AUC, each with 95% confidence intervals computed using the Wilson score interval method (Wilson, 1927). Balanced accuracy, sensitivity, and specificity provide metrics robust to class imbalance, which is relevant here given the unequal group sizes (SCI: $n = 24$; MCI: $n = 46$; ADD: $n = 18$). AUC is invariant to class prevalence and quantifies the probability that the classifier ranks a randomly drawn positive case higher than a randomly drawn negative case. Across the adjacent-stage comparisons, balanced

Table S11. Hedges' g effect sizes and 95% confidence intervals [lower, upper] for the mean group difference of the top-5 discriminative nodes in Nodal Degree (ND), across pairwise comparisons and network types. Positive g: higher value in later-stage group. Negative g: lower value in later-stage group.

Network	Comparison	Node	Mean Diff [95% CI]	Hedges' g
sNET	SCI–MCI	RH_SomMot_16	−1.654 [−2.776, −0.532]	−0.63
		LH_Limbic_TempPole_6	−2.906 [−5.014, −0.798]	−0.60
		LH_Default_PFC_13	−2.543 [−4.406, −0.681]	−0.66
		RH_SomMot_34	−1.359 [−2.366, −0.351]	−0.54
		RH_Vis_19	−1.822 [−3.205, −0.440]	−0.57
	MCI–ADD	LH_Vis_1	2.903 [1.606, 4.201]	1.15
		RH_Vis_28	4.304 [2.179, 6.430]	0.95
		LH_DorsAttn_Post_3	5.256 [2.689, 7.823]	1.07
		LH_Vis_6	2.884 [1.428, 4.341]	0.97
		RH_Vis_26	3.355 [1.606, 5.104]	0.86
fNET	SCI–MCI	LH_SomMot_13	−21.946 [−34.189, −9.702]	−0.86
		LH_SomMot_17	−14.888 [−24.290, −5.486]	−0.77
		RH_Cont_pCun_1	−15.167 [−25.941, −4.392]	−0.57
		LH_Default_Temp_8	−13.453 [−23.477, −3.429]	−0.65
		LH_Vis_6	−15.844 [−27.743, −3.945]	−0.63
	MCI–ADD	LH_Vis_10	−28.198 [−42.415, −13.981]	−1.21
		RH_Default_PFCdPFCm_13	−33.234 [−51.522, −14.947]	−1.19
		LH_Vis_11	−28.626 [−44.874, −12.377]	−1.18
		RH_Default_Temp_2	−28.531 [−46.271, −10.792]	−0.97
		RH_SalVentAttn_PrC_1	−28.507 [−46.556, −10.458]	−1.12

accuracy and AUC generally align with raw accuracy, indicating that performance is not primarily driven by exploiting class imbalance. Sensitivity tends to be lower than specificity in the SCI–MCI comparison, consistent with the smaller effect sizes characteristic of early-stage transitions, whereas the SCI–ADD benchmark comparison shows more balanced sensitivity and specificity, reflecting the larger clinical separation between groups.

All reported 5D classification accuracies exceeded the permutation-based null distribution ($p < 0.05$, 1,000 iterations), confirming that the observed performance reflects genuine discriminative signal rather than chance. The 95% confidence intervals are, however, notably wide, particularly for the MCI–ADD comparison, reflecting the modest cohort sizes in the current study. The width of these intervals underscores that absolute accuracy point estimates should not be over-interpreted, and that future work should prioritise larger sample sizes, particularly for the most clinically relevant MCI and ADD groups.

Table S12. Classification accuracy (Linear SVM, LOOCV) with 95% confidence intervals [lower, upper] for the top-5 nodal feature models (5D), across pairwise group comparisons, network types, and nodal graph metrics.

Network	Comparison	BC	CC	NS	ND
sNET	SCI–MCI	0.73 [0.62, 0.83]	0.76 [0.65, 0.86]	0.71 [0.61, 0.81]	0.66 [0.56, 0.76]
	MCI–ADD	0.83 [0.73, 0.91]	0.69 [0.58, 0.80]	0.64 [0.53, 0.75]	0.72 [0.61, 0.83]
	SCI–ADD	0.81 [0.69, 0.91]	0.88 [0.79, 0.98]	0.88 [0.77, 0.98]	0.79 [0.64, 0.91]
fNET	SCI–MCI	0.74 [0.64, 0.84]	0.79 [0.69, 0.89]	0.69 [0.57, 0.79]	0.59 [0.47, 0.70]
	MCI–ADD	0.83 [0.73, 0.92]	0.70 [0.59, 0.81]	0.78 [0.67, 0.88]	0.81 [0.70, 0.91]
	SCI–ADD	0.79 [0.64, 0.91]	0.74 [0.60, 0.86]	0.79 [0.67, 0.91]	0.83 [0.71, 0.93]

Table S13. Balanced Accuracy and 95% confidence intervals [lower, upper] for the Linear SVM classifier using top-5 nodal features, across all pairwise group comparisons, network types, and nodal graph metrics. Balanced accuracy is the average of sensitivity and specificity, providing a metric robust to class imbalance.

Network	Comparison	BC	CC	NS	ND
sNET	SCI–MCI	0.76 [0.66, 0.87]	0.78 [0.66, 0.87]	0.74 [0.64, 0.84]	0.70 [0.60, 0.80]
	MCI–ADD	0.83 [0.71, 0.92]	0.66 [0.53, 0.79]	0.67 [0.54, 0.78]	0.72 [0.60, 0.84]
	SCI–ADD	0.83 [0.72, 0.92]	0.88 [0.77, 0.97]	0.88 [0.76, 0.97]	0.78 [0.65, 0.90]
fNET	SCI–MCI	0.75 [0.65, 0.85]	0.79 [0.68, 0.88]	0.70 [0.59, 0.80]	0.59 [0.47, 0.70]
	MCI–ADD	0.83 [0.72, 0.92]	0.66 [0.53, 0.79]	0.76 [0.64, 0.87]	0.80 [0.68, 0.91]
	SCI–ADD	0.78 [0.65, 0.90]	0.73 [0.59, 0.85]	0.78 [0.65, 0.90]	0.83 [0.71, 0.93]

Table S14. Sensitivity (true positive rate) and 95% confidence intervals [lower, upper] for the Linear SVM classifier using top-5 nodal features. Sensitivity measures the proportion of true positives correctly identified (the later or more severe clinical group in each comparison).

Network	Comparison	BC	CC	NS	ND
sNET	SCI–MCI	0.65 [0.50, 0.78]	0.72 [0.59, 0.85]	0.65 [0.52, 0.78]	0.57 [0.41, 0.70]
	MCI–ADD	0.83 [0.67, 1.00]	0.61 [0.39, 0.83]	0.72 [0.50, 0.89]	0.72 [0.50, 0.89]
	SCI–ADD	0.71 [0.54, 0.88]	0.88 [0.75, 1.00]	0.92 [0.79, 1.00]	0.79 [0.63, 0.92]
fNET	SCI–MCI	0.72 [0.59, 0.85]	0.78 [0.67, 0.89]	0.65 [0.52, 0.78]	0.59 [0.44, 0.72]
	MCI–ADD	0.83 [0.67, 1.00]	0.55 [0.33, 0.77]	0.72 [0.50, 0.94]	0.78 [0.56, 0.94]
	SCI–ADD	0.79 [0.58, 0.96]	0.79 [0.63, 0.94]	0.83 [0.67, 0.96]	0.88 [0.75, 1.00]

Table S15. Specificity (true negative rate) and 95% confidence intervals [lower, upper] for the Linear SVM classifier using top-5 nodal features. Specificity measures the proportion of true negatives correctly identified (the earlier or less severe clinical group in each comparison).

Network	Comparison	BC	CC	NS	ND
sNET	SCI–MCI	0.88 [0.75, 1.00]	0.83 [0.67, 0.96]	0.83 [0.67, 0.96]	0.83 [0.67, 0.96]
	MCI–ADD	0.83 [0.72, 0.94]	0.72 [0.59, 0.85]	0.61 [0.48, 0.76]	0.72 [0.59, 0.85]
	SCI–ADD	0.94 [0.83, 1.00]	0.89 [0.72, 1.00]	0.83 [0.67, 1.00]	0.78 [0.56, 0.94]
fNET	SCI–MCI	0.79 [0.63, 0.96]	0.79 [0.63, 0.96]	0.75 [0.58, 0.92]	0.58 [0.38, 0.79]
	MCI–ADD	0.83 [0.72, 0.94]	0.76 [0.63, 0.87]	0.80 [0.70, 0.91]	0.83 [0.70, 0.94]
	SCI–ADD	0.78 [0.56, 0.94]	0.67 [0.44, 0.89]	0.72 [0.50, 0.94]	0.78 [0.56, 0.94]

Table S16. Area under the receiver-operating characteristic curve (AUC) and 95% confidence intervals [lower, upper] for the Linear SVM classifier using top-5 nodal features. AUC quantifies the probability that the classifier will rank a randomly selected positive case higher than a randomly selected negative case, and is invariant to class prevalence.

Network	Comparison	BC	CC	NS	ND
sNET	SCI–MCI	0.85 [0.75, 0.95]	0.85 [0.74, 0.94]	0.87 [0.77, 0.95]	0.78 [0.66, 0.88]
	MCI–ADD	0.92 [0.84, 0.98]	0.73 [0.60, 0.85]	0.81 [0.69, 0.91]	0.85 [0.73, 0.94]
	SCI–ADD	0.90 [0.78, 0.98]	0.95 [0.88, 1.00]	0.93 [0.85, 0.99]	0.86 [0.73, 0.95]
fNET	SCI–MCI	0.82 [0.71, 0.91]	0.83 [0.73, 0.92]	0.77 [0.65, 0.88]	0.67 [0.53, 0.79]
	MCI–ADD	0.91 [0.83, 0.97]	0.73 [0.60, 0.86]	0.84 [0.72, 0.94]	0.85 [0.74, 0.94]
	SCI–ADD	0.81 [0.66, 0.94]	0.84 [0.69, 0.96]	0.79 [0.64, 0.92]	0.89 [0.76, 0.99]

7 COMPARISON WITH L2-REGULARISED LOGISTIC REGRESSION

Table S17 reports classification accuracies for L2-regularised logistic regression alongside the primary linear SVM results. The two classifiers produce broadly comparable performance across most settings, supporting the conclusion that discriminative signal resides in the selected nodal features rather than in classifier-specific properties. Where logistic regression falls short of the SVM,

most notably in the sNET SCI-MCI comparison, this likely reflects the sensitivity of maximum-likelihood estimation to the small and imbalanced group sizes in this cohort; the SVM's margin-based objective provides implicit regularisation that is better suited to these conditions. Linear SVM was therefore retained as the primary classifier.

Table S17. Classification accuracy comparison between Linear SVM and L2-regularised Logistic Regression (LR) using top-5 nodal features under identical LOOCV and within-fold feature selection procedures. Values reported as SVM / LR accuracy. †SCI–ADD is a methodological benchmark only; see Section 3.5 of the main manuscript. BC: betweenness centrality; CC: clustering coefficient; NS: nodal strength; ND: nodal degree.

Network	Comparison	BC	CC	NS	ND
sNET	SCI–MCI	0.73 / 0.60	0.76 / 0.69	0.71 / 0.76	0.66 / 0.69
	MCI–ADD	0.83 / 0.73	0.69 / 0.67	0.64 / 0.72	0.72 / 0.73
	SCI–ADD†	0.81 / 0.81	0.88 / 0.81	0.88 / 0.83	0.79 / 0.81
fNET	SCI–MCI	0.74 / 0.74	0.79 / 0.71	0.69 / 0.69	0.59 / 0.60
	MCI–ADD	0.83 / 0.83	0.79 / 0.83	0.78 / 0.78	0.81 / 0.75
	SCI–ADD†	0.79 / 0.76	0.74 / 0.76	0.79 / 0.79	0.83 / 0.81

REFERENCES

[Dataset] (2019). Freesurfer toolbox. `https://surfer.nmr.mgh.harvard.edu/`. Accessed: 2024-09-06

[Dataset] (2019). Fsl toolbox. `https://fsl.fmrib.ox.ac.uk/fsl/fslwiki/FSL`. Accessed: 2024-09-06

[Dataset] (2019). Human connectome project pipeline scripts. `https://github.com/Washington-University/HCPpipelines`. Accessed: 2024-09-06

[Dataset] (2019). Tortoise toolbox. `https://tortoise.nibib.nih.gov/`. Accessed: 2024-09-06

[Dataset] (2024). Recon-all steps. `https://surfer.nmr.mgh.harvard.edu/fswiki/recon-all`. Accessed: 2024-09-06

Coombes, S. A. et al. (2018). Reproducibility of structural brain connectivity and network metrics using probabilistic diffusion tractography. *Scientific Reports* 8, 11562. doi:10.1038/s41598-018-29943-0. The connectivity threshold of 0.01 is recommended for stable graph-metric estimation on normalised structural connectomes.

Fonov, V., Evans, A., Botteron, K., Almli, C., Mckinstry, R., and Collins, L. (2011). Unbiased average age-appropriate atlases for pediatric studies. *NeuroImage* 54, 313–327

Glasser, M. F., Sotiropoulos, S. N., Wilson, J. A., Coalson, T. S., Fischl, B., Andersson, J. L., et al. (2013). The minimal preprocessing pipelines for the human connectome project. *NeuroImage* 80, 105–124

Greve, D. N. and Fischl, B. (2009). Accurate and robust brain image alignment using boundary-based registration. *NeuroImage* 48, 63–72

Smith, S. M. and Brady, J. M. (1997). Susan—a new approach to low level image processing. *International Journal of Computer Vision* 23, 45–78

Wilson, E. B. (1927). Probable inference, the law of succession, and statistical inference. *Journal of the American Statistical Association* 22, 209–212. doi:10.1080/01621459.1927.10502953
